# Non‐Invasive Diagnosis of Moyamoya Disease Using Serum Metabolic Fingerprints and Machine Learning

**DOI:** 10.1002/advs.202405580

**Published:** 2024-12-31

**Authors:** Ruiyuan Weng, Yudian Xu, Xinjie Gao, Linlin Cao, Jiabin Su, Heng Yang, He Li, Chenhuan Ding, Jun Pu, Meng Zhang, Jiheng Hao, Wei Xu, Wei Ni, Kun Qian, Yuxiang Gu

**Affiliations:** ^1^ Department of Neurosurgery Huashan Hospital of Fudan University Shanghai 200040 P. R. China; ^2^ Neurosurgical Institute of Fudan University Shanghai 201107 P. R. China; ^3^ Department of Traditional Chinese Medicine RenJi Hospital School of Medicine Shanghai Jiao Tong University Shanghai 200127 P. R. China; ^4^ School of Biomedical Engineering Institute of Medical Robotics and Med‐X Research Institute Shanghai Jiao Tong University Shanghai 200030 P. R. China; ^5^ State Key Laboratory for Oncogenes and Related Genes Division of Cardiology Renji Hospital School of Medicine Shanghai Jiao Tong University 160 Pujian Road Shanghai 200127 P. R. China; ^6^ Department of Neurosurgery Liaocheng People's Hospital Shandong 252000 China; ^7^ Department of Neurosurgery The First Affiliated Hospital of Fujian Medical University Fujian 350000 China

**Keywords:** biomarkers, fingerprints, mass spectrometry, moyamoya disease diagnosis

## Abstract

Moyamoya disease (MMD) is a progressive cerebrovascular disorder that increases the risk of intracranial ischemia and hemorrhage. Timely diagnosis and intervention can significantly reduce the risk of new‐onset stroke in patients with MMD. However, the current diagnostic methods are invasive and expensive, and non‐invasive diagnosis using biomarkers of MMD is rarely reported. To address this issue, nanoparticle‐enhanced laser desorption/ionization mass spectrometry (LDI MS) was employed to record serum metabolic fingerprints (SMFs) with the aim of establishing a non‐invasive diagnosis method for MMD. Subsequently, a diagnostic model was developed based on deep learning algorithms, which exhibited high accuracy in differentiating the MMD group from the HC group (AUC = 0.958, 95% CI of 0.911 to 1.000). Additionally, hierarchical clustering analysis revealed a significant association between SMFs across different groups and vascular cognitive impairment in MMD. This approach holds promise as a novel and intuitive diagnostic method for MMD. Furthermore, the study may have broader implications for the diagnosis of other neurological disorders.

## Graphical abstract

Moyamoya disease (MMD) increases stroke risk, but current invasive diagnostic methods are costly. To address this, nanoparticle‐enhanced laser desorption/ionization mass spectrometry was used to analyze serum metabolic fingerprints and developed a deep learning diagnostic model, achieving high accuracy in distinguishing MMD patients from healthy controls (AUC = 0.958). This approach holds promise as a novel and intuitive diagnostic method for MMD.

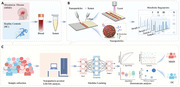

## 1 Introduction

Moyamoya disease (MMD) is a chronic progressive cerebrovascular disorder characterized by bilateral occlusion or stenosis of the internal carotid artery at the entrance to the Willis circle.^[^ ^1^, ^2^ ^]^ It is highly prevalent among people of Asian countries, particularly Korea, Japan, and China, with the annual incidence rising yearly.^[^ ^3^, ^4^ ^]^ The disease primarily manifests as intracranial ischemia and hemorrhage, maintaining a bimodal age distribution with peaks at ages 5 and 40.^[^ ^5^, ^6^, ^7^ ^]^ Among European Caucasian MMD patients, 81% report ischemic symptoms at the initial stage, while 8.5% experience hemorrhagic symptoms.^[^ ^8^ ^]^ The 5‐year stroke risk, encompassing both ischemic and hemorrhagic manifestations, ranges from 40% to 65% based on natural history studies of MMD patients.^[^ ^9^, ^10^ ^]^ Pediatric patients are more prone to intracranial ischemia, while intracranial hemorrhage typically manifests after 25 years of age, leading to varied neurological symptoms depending on the bleeding site.^[^ ^11^, ^12^ ^]^ Rebleeding symptoms, affecting ≈40% of patients, are a significant cause of mortality, with death rates reaching 28.6%.^[^ ^13^ ^]^ Beyond these physical symptoms, MMD patients frequently suffer from cognitive dysfunction, likely attributable to brain structure damage and hypoperfusion.^[^ ^14^ ^]^ Initial studies suggested that 23%–31% of patients experience significant cognitive impairment.^[^ ^15^, ^16^, ^17^ ^]^ However, recent research indicates that 79% of patients exhibit impairment in one or more cognitive domains, with 58% affected in two or more areas.^[^ ^18^ ^]^ MMD is associated with a broad spectrum of cognitive impairment, with severe damage observed in working memory, attention, and executive function.^[^ ^19^ ^]^ Neurocognitive dysfunction induced by MMD, as significant as stroke events, markedly diminishes quality of life and exacerbates economic burdens.^[^ ^20^ ^]^

Timely surgical intervention not only is an effective treatment for reducing the long‐term stroke risk but also can reverse hypoperfusion and mitigate brain microstructure damage, underscoring the importance of early detection and intervention in improving MMD outcomes.^[^ ^21^, ^22^, ^23^ ^]^ Digital subtraction angiography (DSA) remains the diagnostic gold standard for MMD,^[^ ^24^, ^25^ ^]^ with an AUC of 0.813 in DSA image models.^[^ ^26^ ^]^ Despite its time‐consuming nature and lower spatial resolution, magnetic resonance imaging/angiography (MRI/MRA) is increasingly used to identify the disease's major neuroradiological features.^[^ ^27^ ^]^ These procedures, however, are invasive, expensive, and require expert annotation of images, making them unsuitable for mass screening. The advent of non‐invasive, low‐cost blood tests may revolutionize MMD detection.

Blood tests are indispensable for the early diagnosis of diseases as they encompass a plethora of biomarkers capable of providing disease‐related information, containing metabolic byproducts, inflammation markers, biochemical indicators, immunoserum markers, and hormone levels.^[^ ^28^, ^29^, ^30^ ^]^ In comparison to alternative diagnostic approaches, such as tissue biopsies or imaging tests, blood tests exhibit superior safety, simplicity, and cost‐effectiveness attributable to their non‐invasive nature, convenience, and broad applicability.^[^ ^31^, ^32^, ^33^ ^]^ A solitary blood sample can furnish abundant information, facilitating physicians' assessment of a patient's health status and timely detection of potential diseases. The significance of blood tests in early disease diagnosis extends beyond prevalent conditions like cancer, cardiovascular diseases, and diabetes,^[^ ^34^, ^35^, ^36^ ^]^ encompassing the vital realm of brain diseases.^[^ ^37^ ^]^ Thus, blood tests are capable of uncovering specific genetic variations or distinct biomarkers of brain diseases, offering a dependable basis for diagnosing brain conditions.^[^ ^38^, ^39^ ^]^ In conclusion, blood tests are poised to serve an irreplaceable role in early brain disease detection, their speed, precision, and cost‐efficiency rendering them essential in clinical settings.

Metabolites hold the potential to serve as biomarkers for the diagnosis of brain diseases, given their ability to cross the blood‐brain barrier (BBB).^[^ ^40^ ^]^ This characteristic allows for the acquisition of metabolite information that reflects the state of brain diseases from the blood, obviating the need for more invasive diagnostic procedures such as brain tissue biopsies or neuroimaging.^[^ ^38^, ^41^, ^42^ ^]^ Furthermore, disease conditions can impact the metabolic activities of brain cells, leading to changes in metabolite levels.^[^ ^43^ ^]^ The pathophysiological processes underlying brain diseases can be elucidated by measuring these alterations, aiding in early diagnosis and monitoring of disease progression. Serum metabolic fingerprints (SMFs), a type of blood test that employs small molecule metabolites (such as amino acids, peptides, lipids), have the capacity to identify specific diseases by analyzing metabolite patterns in the blood.^[^ ^35^, ^44^, ^45^, ^46^ ^]^ This non‐invasive method of testing, characterized by its convenience, speed, and cost‐effectiveness, can be widely applied in clinical practice and has been reported in intracranial diseases, including stroke,^[^ ^36^ ^]^ acute traumatic brain injury,^[^ ^47^ ^]^ intracranial aneurysm,^[^ ^48^ ^]^ and other diseases.^[^ ^45^ ^]^ Consequently, metabolic diagnosis plays a crucial role in MMD diagnosis, as it can provide information about the brain's microenvironment and disease state, offering support for early diagnosis, treatment selection, and disease monitoring.

Over the last ten years, analytical methods, including nuclear magnetic resonance (NMR) and mass spectrometry (MS), have emerged in the field of SMFs.^[^ ^49^, ^50^, ^51^, ^52^, ^53^ ^]^ NMR detection has limitations in sensitivity, molecular recognition, and lengthy detection times due to relaxation.^[^ ^54^ ^]^ MS, measuring the mass‐to‐charge ratio (m/s), is traditionally used with chromatography for sample purification and metabolite enrichment, which can limit analytical efficiency and throughput.^[^ ^44^, ^55^, ^56^, ^57^ ^]^ In contrast, nanoparticle‐assisted laser desorption/ionization (LDI MS) offers fast analysis (approximately seconds), low sample volume (0.1–1 µL), simple sample preprocessing, high sensitivity, and low cost.^[^ ^55^, ^58^, ^59^ ^]^ It presents a high‐performance technique that could significantly aid SMFs analysis of MMD. Concurrently, machine learning unveils correlations and mechanisms between metabolomics and diseases, offering a novel perspective for disease comprehension and treatment.^[^ ^60^, ^61^ ^]^ Machine learning algorithms are capable of identifying biomarkers linked to specific diseases, thereby enabling early disease detection and diagnosis.^[^ ^45^, ^62^, ^63^, ^64^, ^65^ ^]^ Moreover, machine learning can forecast disease risk and progression using patient metabolomic data through classification and predictive models, providing a basis for treatment selection in personalized medicine.^[^ ^36^, ^66^ ^]^ Nonetheless, challenges pertaining to model interpretability and generalizability must be addressed to improve the reliability and precision of machine learning in metabolomics‐assisted diagnosis.

**Scheme 1:**
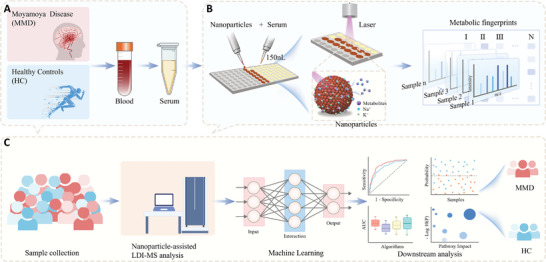
Schematics for MMD diagnosis based on SMFs and machine learning. A) Sample collection: collection of serum samples from the MMD group and HC group. B) Nanoparticle‐assisted LDI MS analysis: serum samples were arrayed on chip, followed by nanoparticles to obtain raw mass spectra and serum metabolic fingerprints. C) Processing flow: The MMD group and HC group were differentiated after conducting machine learning of serum metabolic fingerprints (SMFs) for signal selection.

In our study, we employed nanoparticle‐assisted LDI MS for the extraction of serum metabolic fingerprints (SMFs) from MMD group, aiming for non‐invasive diagnosis. Initially, we procured SMFs from 288 serum samples (comprising 144 MMD and 144 HC), as depicted in **Scheme** **1**A, demonstrating high reproducibility and low sample volume (as shown in Scheme 1B). Utilizing machine learning, we distinguished the MMD group from the HC group, achieving an optimal area under the curve (AUC) of 0.958 (as illustrated in Scheme 1C). A cluster analysis was performed on the group of MMD patients based on metabolites to examine the variations in cognitive impairment levels across groups. Our study has propelled the analysis of serum metabolism in MMD forward and laid the groundwork for future non‐invasive blood tests for MMD.

## 2 Results

### 2.1 Characterization of MMD Specific SMFs

**Figure 1:**
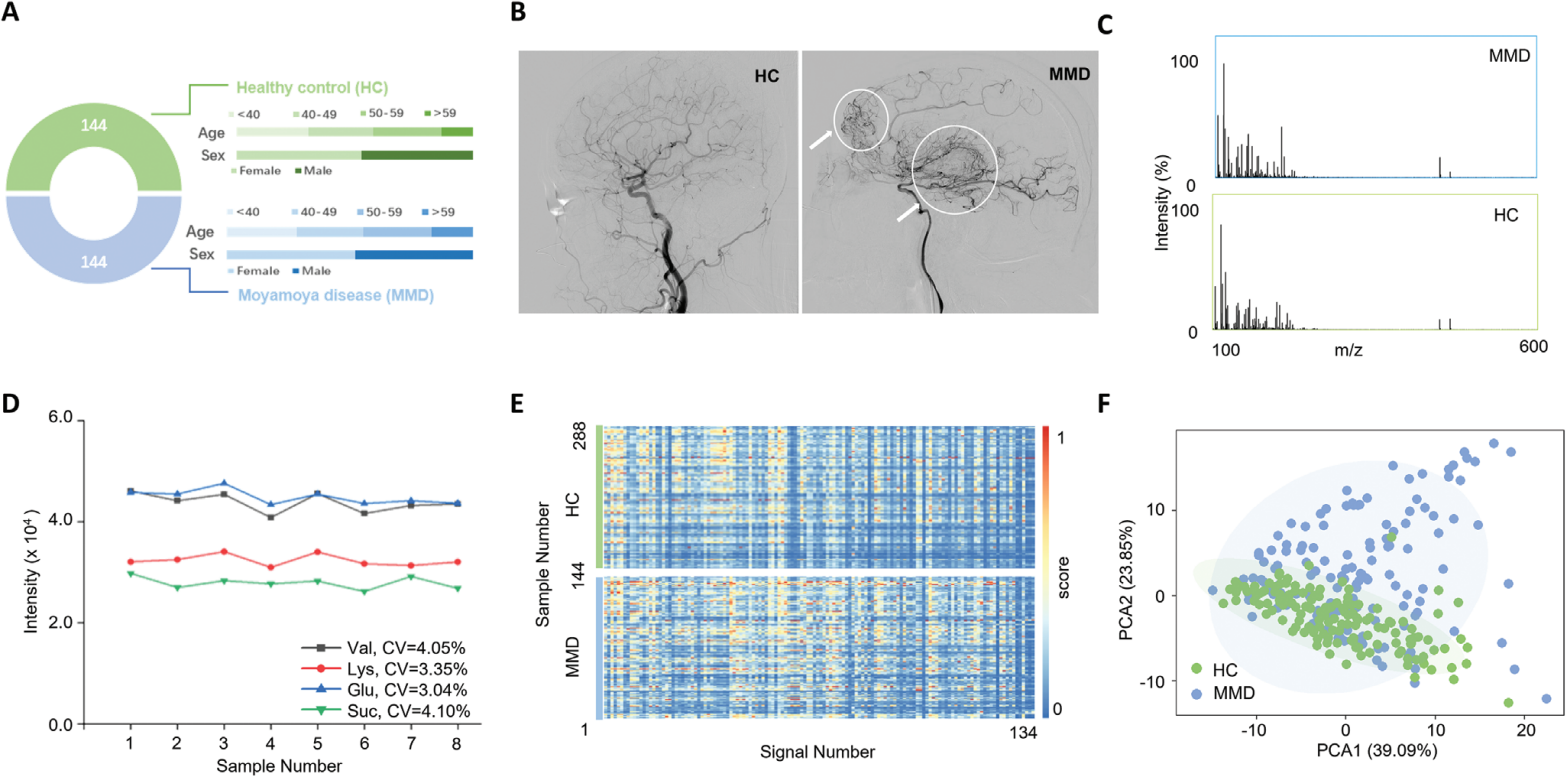
Characterization of MMD‐specific SMFs. A) The distribution of age and sex among the 144 participants in the MMD group and the 144 participants in the HC group. B) A representative DSA image from an MMD patient. C) Two typical mass spectra at the m/z range of 100 to 600 are shown for serum samples of MMD group and HC group. D) Intensities of four molecular peaks (gray line for [Val + Na]^+^ at an m/z of 140.14, red line for [Lys + Na]^+^ at an m/z of 169.09, blue line for [Glu + Na]^+^ at an m/z of 203.05, green line for [Suc + Na]^+^ at an m/z of 365.11) for eight replicates per sample. E) A heat map of distinct metabolic fingerprints for 288 serum samples was constructed using 134 m/z signals obtained through data preprocessing. F) The PCA plot for the MMD group (depicted as bule points) and HC group (depicted as green points).

For the analysis of MMD‐specific SMFs, we gathered 288 serum samples, comprising 144 from patients diagnosed with MMD and 144 from healthy controls (HC), as depicted in **Figure** **1**A. The diagnosis for the MMD group was confirmed through DSA, with representative DSA images from the MMD group presented in Figure 1B and Figure S1 (Supporting Information). Concurrently, the HC group exhibited no clinical indications of MMD or any other significant diseases. Comparative analysis between the MMD and HC groups indicated no significant differences in age, gender, or Body Mass Index (BMI), with the P‐value for age exceeding 0.5, an F‐value for sex of 0.906, and a P‐value for BMI greater than 0.05 (Table S1, Supporting Information).

We established a serum metabolic fingerprinting database utilizing nanoparticle‐assisted LDI MS analysis, which exhibited high reproducibility and necessitated minimal sample volumes with optimal nanoparticles (Figure S2, Supporting Information). To ascertain the optimal dilution factor, we procured the mass spectrum of the undiluted serum, along with the serum diluted 20‐fold, 10‐fold, 5‐fold, and undiluted. The 10‐fold diluted serum exhibited the most optimal mass spectrum, with significant intensity differences and more peaks with signal‐to‐noise ratios (S/N) exceeding 3 (Figure S3, Supporting Information). For stability assessment, we collected mass spectra from standard serum on days 1, 3, 5, and 7, with no clear distinction between the four‐day results and similarity analyses showing more than 96% of scores exceeded 0.9 (Figure S4, Supporting Information). Regarding detection sensitivity, the efficient absorption and transfer of nanoparticle‐assisted LDI MS resulted in enhanced detection sensitivity compared to organic matrices (Figure S5, Supporting Information).

Conventional methods like liquid chromatography‐mass spectrometry (LC‐MS) and gas chromatography‐mass spectrometry (GC‐MS) necessitate tedious sample pretreatment and prolonged chromatographic separation (≈40 min) to diminish the complexity of biological samples and enrich the molecules.^[^ ^67^, ^68^ ^]^ Contrastingly, nanoparticle‐assisted LDI MS facilitates size‐selective capture of metabolites and in‐source cation adduction (e.g., Na^+^ and K^+^) for in‐situ pre‐concentration.^[^ ^35^, ^69^, ^70^ ^]^ This allows for the direct detection of metabolites in complex biological fluids with minimal biological samples (0.1 – 1 µL). Moreover, we attained high analysis speeds (≈5 – 20 s per sample) owing to the 2000 laser emission at a pulse frequency of 1000 Hz when detected by chip microarrays and nanoparticle‐assisted LDI MS devices.^[^ ^44^, ^46^ ^]^ This satisfies the requirements for high‐throughput detection and provides a high‐performance platform for disease diagnosis through body fluids, such as blood and urine.

Our methodology exhibited high analytical throughput, generating ample m/z signals for each metabolic fingerprint. In each sample, we procured ≈124 000 data points in the original MS result, with over 98% of strong m/z signals acquired within the low mass range (m/z of 100 to 600) (Figure 1C; Figure S6A, Supporting Information). Additionally, the intensity CVs of the four molecular peaks ([Val + Na]^+^ at an m/z of 140.14, red line for [Lys + Na]^+^ at an m/z of 169.09, blue line for [Glu + Na]^+^ at an m/z of 203.05, green line for [Suc + Na]^+^ at an m/z of 365.11) in the standard ranged from 3.04% to 4.10% (Figure 1D), indicating the reliability of SMFs and their potential for further diagnostic applications.

After data preprocessing, which encompassed binning, smoothing, baseline correction, peak detection, and alignment, we extracted the MMD‐specific SMFs containing 134 m/z signals from the original raw mass spectra (Figure 1E). To reduce bias, each sample was measured five times, using the average to represent the SMFs of the sample. We selected 10 m/z values to calculate the relative standard deviation (RSD), which was found to be less than 15% (Figure S6C, Supporting Information). Furthermore, each batch experiment included five quality control (QC) samples, and we observed metabolic clustering with the standard serum collected in each independent experiment batch, indicating data reliability (Figure S6B, Supporting Information). Meanwhile, the similarity scores for these batches surpassed 0.95, indicating the data's stability and reliability (Figure S6D, Supporting Information). Principal component analysis (PCA) revealed a degree of separation between the MMD and HC group (Figure 1F). These findings suggest that the MMD‐specific SMFs, based on nanoparticle‐assisted LDI MS, can be utilized in the construction of the MMD diagnosis model and signal selection, thereby achieving the capability to distinguish the MMD group from the HC group.

### 2.2 Construction of a SMFs‐Based Diagnosis Model for MMD

To complement the diagnosis of imaging methods, we verified that the metabolite information of SMFs, consisting of 134 signals, showed a promising future as a blood examination method to distinguish MMD group from HC group. As serum samples were randomly assigned to discovery cohorts of *n* = 230 (*n* = 115 MMD and *n* = 115 HC) and *n* = 58 independent validation cohorts (*n* = 29 MMD and *n* = 29 HC), we constructed a MMD diagnosis model using machine learning based on MMD specific SMFs. At the outset, power analysis was conducted to determine the required sample size for the experiment, targeting a false discovery rate (FDR) of 0.1. With a sample size of 230 (115/115, MMD/HC), a power level of 0.90 can be reached. (Figure S6E, Supporting Information).

**Figure 2:**
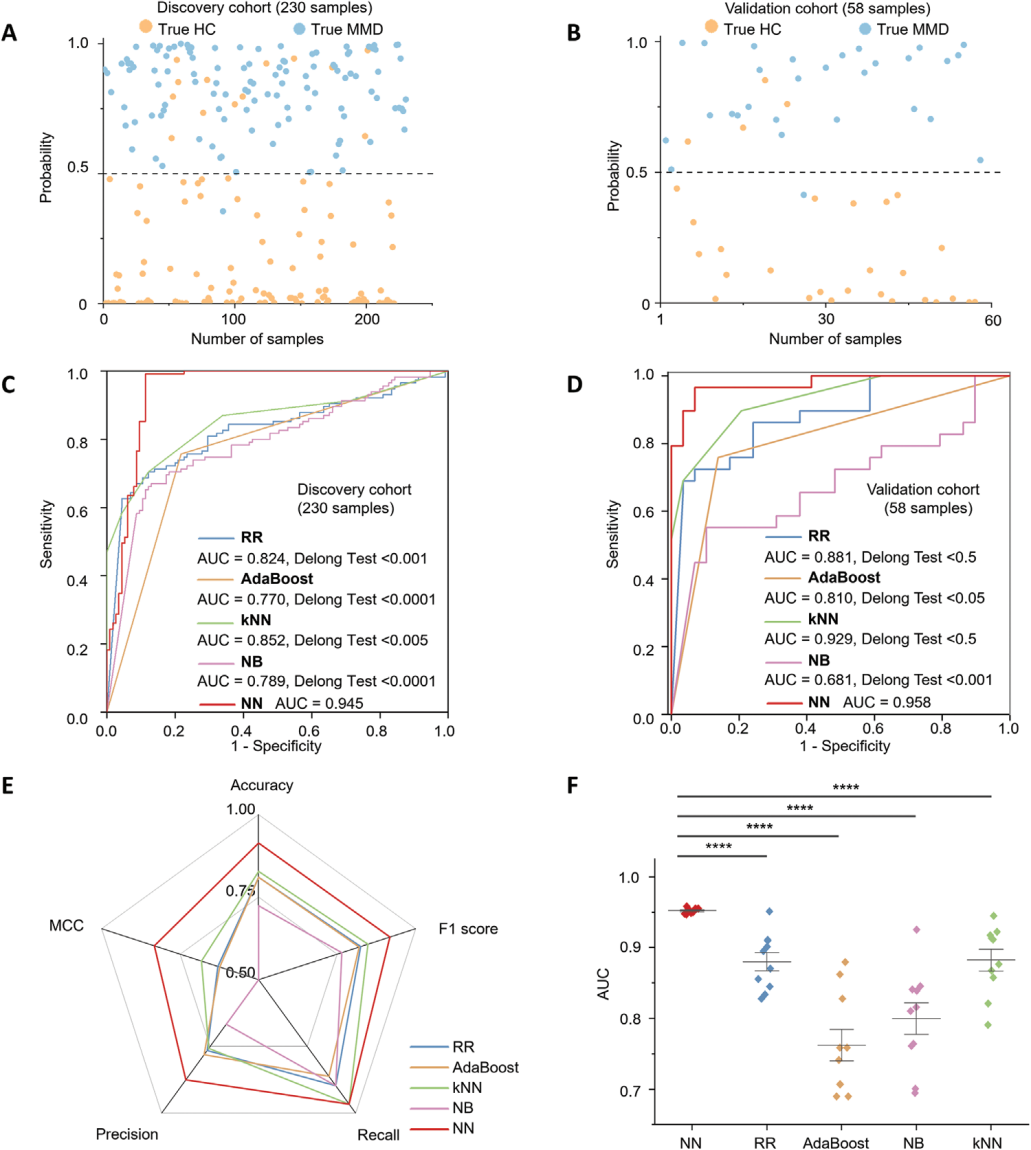
The machine learning model for MMD screening. A sample‐level scatter plot stratifies the HC group (depicted in orange) and the MMD group (depicted in blue) for A) discovery cohort (n = 230; 115/115, HC/MMD) and B) validation cohort (n = 58; 29/29, HC/MMD). The ROC curves for five typical algorithms (NN, RR, AdaBoost, kNN, and NB) for diagnosing the MMD group from the HC group in C) the discovery cohort and D) the validation cohort. E) Model evaluation of five algorithms, including accuracy, F1 score, recall, precision, and MCC. F) AUC value of 10 repeated instances for the five algorithms in the validation cohort.

Machine learning has been integrated with metabolomics data to diagnose various diseases, including stroke,^[^ ^36^ ^]^ breast cancer,^[^ ^71^ ^]^ stomach cancer,^[^ ^72^ ^]^ metabolic syndrome,^[^ ^73^ ^]^ and cerebral aneurysm.^[^ ^48^ ^]^ This integration allows for efficient analysis of metabolomic data, identifying potential diagnostic markers for clinical use. To test the classification performance of MMD specific SMFs, five algorithms (Neural Network [NN],^[^ ^36^, ^74^ ^]^ Adaptive Boosting [AdaBoost,^[^ ^75^ ^]^] Ridge Regression [RR,^[^ ^76^ ^]^] K‐Nearest Neighbor [kNN]^[^ ^77^ ^]^ and Naïve Bayes [NB]^[^ ^78^ ^]^), which were suitable for disease diagnosis and classification,^[^ ^73^, ^79^, ^80^ ^]^ were chosen for model construction. We verified the effectiveness of the model using 10‐fold cross‐validation, while evaluating the average performance of the model by sensitivity, specificity, and AUC. For the discovery cohort, we achieved AUC of 0.945 (95% CI 0.912 to 0.977, **Figure** **2**A,C) with NN algorithm. For comparison, RR showed AUC with 0.824 (95% CI 0.767 to 0.880, Figure S7A, Supporting Information), AUC with 0.770 (95% CI 0.707 to 0.833, Figure S7B, Supporting Information) was provided by AdaBoost. Meanwhile, kNN (AUC = 0.852, 95% CI 0.801 to 0.903, Figure S7C, Supporting Information) and NB (AUC = 0.789, 95% CI 0.729 to 0.849, Figure S7D, Supporting Information) (Table S2, Supporting Information) were assessed. Notably, NN showed optimization performance in all five algorithms (p < 0.05, DeLong test, Table S3, Supporting Information), though the AUC of other algorithms differentiating MMD group from HC group in the discovery cohort were greater than 0.750. Furthermore, we obtained consistent results in the independent validation cohort in the blind test, with an AUC value of 0.958 (95% CI 0.911 to 1.000, Figure 2B,D; Figure S8, Supporting Information) for the NN.

Moreover, we compared the five algorithms in more details including accuracy, F1 score, recall, precision, and stability to further illustrate the superiority of NN algorithm. Compared with the other four algorithms, the NN algorithm performed best in accuracy (0.914), F1 score (0.918), MCC (0.832), recall (0.966), and precision (0.875) (Figure 2E; Table S4, Supporting Information), whose performance was greater than other algorithms significantly. Moreover, the AUC value obtained by 10 repeated tests in the validation cohort was carried out. Obviously, the average AUC value of NN was remarkably better than that of other 4 algorithms, and the performance was the most stable as the AUC values were highly consistent across 10 repetitions (Figure 2F; Table S5, Supporting Information). Also, through 20 repetitions at 10‐fold and AUC value undergoing 20‐fold in validation cohort, the stability of the NN model results has been further verified (Figure S9, Supporting Information). Notably, NN algorithm has significantly higher performance in MMD specific SMFs to make a distinction between MMD group and HC group.

Compared with commonly used traditional machine learning algorithms, end‐to‐end learning and multilayer stacked networks contribute to the excellent performance of NN algorithms.^[^ ^36^ ^]^ When traditional machine learning algorithms need to gradually integrate separate signal extraction and supervised classification processes into reliable routines, end‐to‐end learning in neural networks combines signal extraction and disease classification into a single step. This involves computing convolution and nonlinear transformations of features to extract high‐level abstractions for final classification within the network.^[^ ^81^, ^82^ ^]^ For multilayer stacked networks, locally connected 1D layers and stacked nonlinear signal interaction layers are built in NN to solve linear and nonlinear problems between input signals. Compared to multilayer perception, the nonlinear signal interaction layer, in particular, prevents potential overfitting and enhances classification performance in scenarios with limited samples. In comparison, traditional machine learning algorithms such as RR, AdaBoost, kNN, and NB, while capable of addressing certain nonlinear complexities, exhibit limited performance in terms of scalability and flexibility, and are sensitive to noise and outliers in the data.

### 2.3 Identification of a Metabolic Biomarker Panel

**Figure 3:**
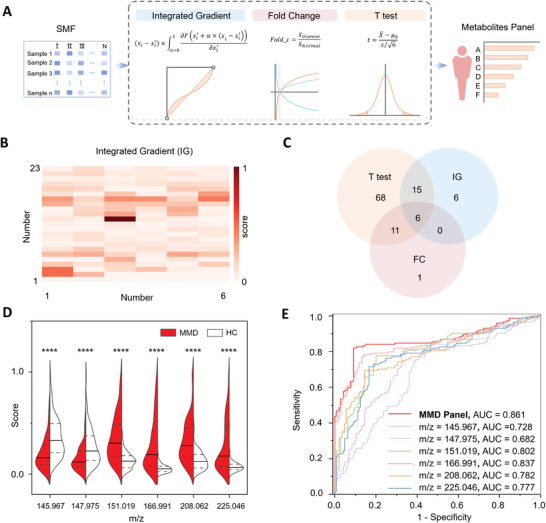
Biomarker panel development. A) The procedure for deriving metabolite panels via three methods based on SMFs. B) A heatmap representing the contribution value of 134 signals in IG. C) The intersection of the signals filtered by the three methods, including integrated gradients (IG), fold change (FC), and T test. D) The violin plot illustrated the differential expression of six metabolites between the MMD group (depicted in red) and HC group (depicted in white), with P‐value (^****^ *p* < 0.0001) indicated at the top of each violin plot. E) The ROC curves exhibit a higher AUC of 0.861 using the metabolic biomarker panel compared to a single metabolic biomarker (AUC ranging from 0.682 to 0.837).

To further identify the characteristic signals associated with MMD, we screened three aspects: integrated gradients (IG), fold change (FC), and T test. This allowed us to obtain the MMD‐specific metabolite panel (**Figure** **3**A). Integrated Gradients (IG) is a method for interpreting the predictions of deep learning models by determining how much each signal contributes to a model.^[^ ^83^, ^84^ ^]^ In this regard, we selected the top 20% signals based on their contribution degree in the NN algorithm (Figure 3B). Simultaneously, we compared the SMFs of the MMD group and HC group to identify the characteristic signals with |Log_2_(FC)| greater than 1.8 and a P‐value less than 0.05 (Figure S10A, Supporting Information). Furthermore, by matching in the HMDB database, we cataloged 134 potential metabolites in Table S6 (Supporting Information), and from this, we identified 6 metabolites that constitute a specific panel for MMD (Figure 3C). These metabolites exhibited significant differences in the serum between the MMD group and HC group, with 2 metabolites down‐regulated and 4 metabolites up‐regulated in the MMD group (Figure 3D). To confirm the stability of these six serum metabolites, we utilized Fourier Transform Ion Cyclotron Resonance (FT ICR) mass spectrometry and LC MS, which detected these metabolites with absolute molecular weight deviations under 150 ppm (Table S7, Supporting Information).

To assess the predictive power of the MMD‐specific panel consisting of 6 metabolites, we utilized the MMD diagnosis model to calculate the AUC value. The panel exhibited an improved diagnostic AUC (0.861), surpassing that of a single metabolic biomarker (AUC value of 0.682–0.837) (Figure 3E; Figure S10B and Table S8, Supporting Information). The performance of the MMD‐specific panel suggests its potential as a promising biomarker for MMD clinic diagnosis. Additionally, we conducted a search for metabolites associated with brain diseases such as epilepsy and Alzheimer's, which have been previously reported in HMDB, using the m/z values of metabolic signals (Tables S6 and S7, Supporting Information).^[^ ^85^, ^86^ ^]^ Furthermore, to validate the repeatability of the six metabolic indicators, we collected 86 samples from three centers as an independent external validation set with no obvious separation of the three‐center samples (Table S9 and Figure S10C, Supporting Information). The AUC reached 0.867 based on the 6 metabolic signals, with specificity 0.892 and sensitivity 0.735, indicating that the six metabolic indicators can be clinically applied (Figure S10D,E, Supporting Information). Finally, during pathway enrichment analysis using the Kyoto Encyclopedia of Genes and Genomes (KEGG) pathway library, we investigated the potential biological relevance and metabolic pathways involving these six metabolic signals. There are 7 pathways involved, including taurine and hypotaurine metabolism, vitamin B6 metabolism, nicotinate and nicotinamide metabolism, pantothenate and CoA biosynthesis, beta‐Alanine metabolism, pyrimidine metabolism, and primary bile acid biosynthesis (Figure S11A, Supporting Information). Furthermore, the pathways including 1) taurine and hypotaurine metabolism (PI of 0.429), 2) vitamin B6 metabolism (PI of 0.078), and 3) pyrimidine metabolism (PI of 0.053) have a significant pathway impact (PI) > 0.05 and a hit number (the number of matched metabolites in the pathway) ≥1 (Table S10, Supporting Information).

Utilizing biofluid biomarkers for MMD diagnosis could revolutionize clinical practice, particularly as non‐invasive, cost‐effective liquid biopsy techniques like blood tests are currently underexplored in MMD clinical diagnosis. Currently, traditional imaging techniques such as MRI and DSA remain the primary diagnostic tools for MMD, despite their inherent risks, including potential anaphylaxis and nephropathy from contrast medium use.^[^ ^27^ ^]^ Furthermore, MMD diagnosis via imaging necessitates a seasoned physician specializing in intracranial diseases, posing a significant barrier for many patients residing in smaller cities. In contrast, assessing biomarker content via blood tests offers a convenient, non‐invasive, and intuitive method for MMD diagnosis.

Alternatively, few studies focusing on proteomics and genomics analyses have reported some changed protein and nucleic acid caused of MMD.^[^ ^87^, ^88^, ^89^ ^]^ Concurrently, the use of small metabolites in MMD diagnosis is infrequent, largely due to the limited sample size (20–45) in studies. In comparison, our study, based on a well‐structured cohort, demonstrated superior diagnostic performance with an AUC of 0.958 (95% CI of 0.911 to 1.000), sensitivity of 0.966, and specificity of 0.862, indicating promise for MMD diagnosis in clinical settings.

### 2.4 Differentiation of Cognitive Impairment by Metabolic Grouping

**Figure 4:**
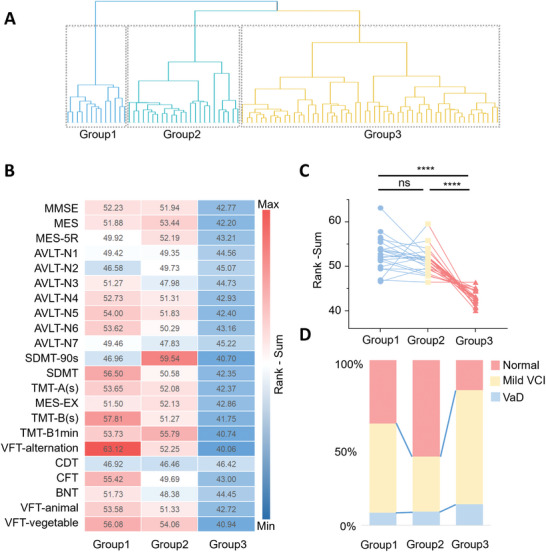
The association between metabolite pattern and cognitive function. A) The outcome of hierarchical clustering analysis. B) The heatmap of the rank‐sum of neuropsychological scores indicates that Group 3 exhibited the poorest performance. C) Group 3 demonstrated the lowest neuropsychological score in comparison to Group 1 (^****^ *p* < 0.0001) and Group 2 (^****^ *p* < 0.0001). D) Group 3 comprised the highest proportion of mild Vascular Cognitive Impairment (VCI) and Vascular Dementia (VaD).

To elucidate the clinical significance of metabolites in MMD, we explored the correlation between metabolite patterns and cognitive functions reflecting MMD severity. Hierarchical clustering analysis revealed a natural division of MMD metabolite patterns into three distinct groups (**Figure** **4**A). We retrospectively assessed neuropsychological performance in 92 patients (Table S11, Supporting Information), evaluating neurocognitive functions including global cognition, verbal memory, attention, executive function, visuospatial ability, and language (Figure S11B,C; Tables S12 and S13, Supporting Information).^[^ ^90^ ^]^ Each neuropsychological scale's scores were represented using rank‐sum. Interestingly, we found that Group 3′s neuropsychological performance was significantly inferior to that of Groups 1 and 2 (Kruskal–Wallis test, *p* < 0.001), with no significant difference observed between Groups 1 and 2 (Figure 4B,C). This difference persisted both in the data of 134 metabolites and on the MMD‐specific panel of 6 metabolites, with Group 3 consistently showing significantly lower neuropsychological performance than Groups 1 and 2 (Figure S12A, Supporting Information). Additionally, a significantly higher proportion of patients in Group 3 were diagnosed with vascular cognitive impairment (VCI), including mild VCI and vascular dementia (VaD), compared to Groups 1 and 2 (Kruskal–Wallis test, *p* = 0.002) (Figure 4D; Figure S12B, Supporting Information). These results suggest that the metabolite patterns in Group 3 are associated with poor neuropsychological performance and VCI.

Currently, VCI diagnosis primarily depends on comprehensive evaluations by professional neuropsychologists, incorporating clinical manifestations, neuropsychological assessments, and neuroimaging findings.^[^ ^91^ ^]^ The early stages of mild VCI can often be elusive without the use of neuropsychological tests. However, the diagnostic efficacy of neuropsychological tests can be influenced by factors such as age,^[^ ^92^ ^]^ educational background,^[^ ^93^ ^]^ cultural differences,^[^ ^94^ ^]^ and test‐retest effects.^[^ ^95^ ^]^ Thus, employing serum biomarkers could offer a promising new approach for screening cognitive impairment. In this study, we discovered a strong correlation among specific MMD metabolite patterns, neuropsychological performance, and the incidence of VCI. The underlying pathophysiological mechanisms remain elusive, potentially involving metabolites released from dysfunctional neurons and/or neuroglial cells, which then traverse the BBB and are detected in peripheral circulation.

To date, several studies have reported potential peripheral biomarkers for identifying VCI. However, most of these studies were conducted between VCI and HC, where significant biases may arise due to primary diseases like stroke or atherosclerosis.^[^ ^96^, ^97^ ^]^ In contrast to those studies, our research was conducted within the MMD group, effectively eliminating bias from primary diseases. These findings offer new insights into the role of serum metabolite biomarkers in identifying VCI in MMD patients, which is strongly associated with MMD severity.

## 3 Conclusion

In summary, we achieved rapid sample detection using the nanoparticle‐assisted LDI MS platform. Coupled with machine learning, we successfully diagnosed MMD patients with an AUC of up to 0.958, and based on this, we screened to obtain MMD‐related metabolic panels. Finally, the cognitive status of the patients with MMD was analyzed. we discovered a strong correlation between specific MMD metabolite patterns, neuropsychological performance, and the incidence of VCI. Our work could potentially contribute to the diagnosis of MMD patients via clinical blood tests. However, there are certain limitations to our work that should be acknowledged. First, it is essential to characterize the compounds and verify their functionality, either in vitro or in vivo. Second, integrating our metabolic panels with imaging to construct a multimodal database could potentially enhance clinical practice.

## 4 Experimental Section

### Chemicals and Reagents

Chemicals and reagents in this work included standard small metabolites reagents to prepare nanoparticles and organic matrices. For standard small metabolites, L‐valine (Val, 98%), D‐glucose (Glu, 99.5%), L‐arginine (Arg, 99.5%), L‐leucine (Leu,98%), Sucrose (99.5%) were purchased from Sigma–Aldrich (St. Louis, MO, USA). For reagents to prepare nanoparticles, trisodium citrate dihydrate (99%), ferric chloride hexahydrate (97%), ethylene glycol (99.5%), and anhydrous sodium acetate (99%) were purchased from Sinopharm Chemical Reagent Beijing Co., Ltd. (Beijing, China). For organic matrices, α‐cyano‐4‐hydroxy‐cinnamic acid (CHCA) and 2,5‐dihydroxybenzoic acid (DHB) were purchased from Bruker (Bremen, Germany). Trifluoroacetic acid (TFA, 99.5%) was purchased from Macklin Biochemical Co., Ltd. (Shanghai, China). Acetonitrile (ACN, 99%) was purchased from Aladdin Reagent (Shanghai, China). LC‐MS grade acetonitrile (ACN), HPLC grade methanol (MeOH) were obtained from Honeywell. (Houston, TA, USA). All chemicals and reagents above were directly used without further purification or enrichment. All aqueous solutions were prepared using deionized water (18.2 MΩ·cm, Milli‐Q, Millipore, GmbH) for all the experiments.

### Clinical Subjects and Sample Harvesting

This study was approved by the institutional review boards of Huashan Hospital, Fudan University (reference number: KY2015‐256 and KY2022‐821), and all participants or their guardians signed the informed consent. 288 blood samples of MMD patients and health controls were harvested between September 2020 and October 2022. All MMD patients underwent Digital subtraction angiography (DSA) to confirm the diagnosis by two senior neurosurgeons. Healthy controls were recruited from the Health Examination Center of Hashan Hospital, Fudan University. Additionally, from November 2023 to January 2024, 86 samples were collected for an external validation cohort, including 49 MMD samples from three centers: Center A (Huashan Hospital), Center B (Liaocheng People's Hospital), and Center C (The First Affiliated Hospital of Fujian Medical University Hospital). Blood samples were obtained at the same time as regular blood tests after overnight fasting and then centrifuged for 10 min (1500 g, 4 °C). Serum samples were aliquoted in sterile centrifuge tubes and stored at −80 °C storage freezer. The results presented are derived solely from all qualified samples suitable for mass spectrometry analysis, and no samples were discarded during the course of the project.

### Assessment of Cognitive Impairment in Clinical Subjects

A battery of neuropsychological assessment and diagnosis of vascular cognitive impairment were performed by three professional neuropsychologists, including Mini‐Mental State Examination (MMSE), Memory and Executive Screening (MES),^[^ ^98^ ^]^ Symbol Digit Modalities Test (SDMT),^[^ ^99^ ^]^ Trail Making Test A and B (TMT‐A and B),^[^ ^100^ ^]^ Chinese auditory verbal learning test (AVLT),^[^ ^101^ ^]^ Animal/Vegetable/Alternation Verbal Fluency Test (VFT),^[^ ^102^ ^]^ Boston Naming Test (BNT),^[^ ^103^ ^]^ Clock Drawing Test (CDT)^[^ ^104^ ^]^ and Rey‐Osterrieth Complex Figure Test (CFT).^[^ ^105^ ^]^ The diagnosis criteria of VCI were based on the Guidelines from the Vascular Impairment of Cognition Classification Consensus Study (VICCCS).^[^ ^91^ ^]^

### Nanoparticle‐Assisted LDI MS Experiments

Nanoparticle‐assisted LDI MS experiments of standard small metabolites and aqueous humor samples of MMD patients were conducted on the Bruker systems with Nd:YAG lasers (355 nm) for Autoflex (Time of Flight‐Mass Spectrometry, TOF‐MS) and Solarix 7.0T (Fourier Transform‐Ion Cyclotron Resonance‐Mass Spectrometry, FT‐ICR‐MS), using prepared nanoparticles and organic matrices for laser desorption/ionization (LDI) process. Typically, the prepared nanoparticles were suspended in deionized water at a 1.0 mg mL^−1^ concentration. The CHCA and DHB were dissolved in 0.3% TFA aqueous solution/ACN (2/1, v/v) to prepare a saturated and 10 mg mL^−1^ solution. In LDI MS experiments, 1 µL of analyte solution (prepared standard small metabolites or samples) was first spotted on the polished steel plate and dried, followed by depositing 1 µL of nanoparticle suspension or organic matrix solution and dried in air at room temperature. Standard small metabolites were utilized for precise mass measurement during mass calibration, and all MS experiments were performed in the positive ion mode. Each analysis was conducted with a pulse frequency of 1000 Hz and 2000 laser shots. The acceleration voltage was set as 20 kV, and the delay time was optimized to 200 ns. For internal quality control in each batch of testing, a nine‐grid spotting method was employed during the sample analysis process. In this 3 × 3 matrix, the center point served as the quality control spot (composed of various standard molecules), where calibration was performed before collecting the metabolic spectra of the other eight samples in the matrix. To address instrument detection biases, each sample point is measured five times in each batch test, and the average of these five measurements is used to obtain stable spectral values for further analysis.

### LC‐MS Experiments

To prepare serum, 400 µL of a methanol: acetonitrile mixture (1:1 v:v) was added to a 100 µL serum sample and vortexed for 60 s. The mixture was then incubated at −20 °C for 2 h to facilitate protein precipitation. Following incubation, the sample was centrifuged at 13 000 rpm for 15 min at 4 °C, and the supernatant was collected and evaporated to dryness using a vacuum concentrator. The dry extracts were subsequently resuspended in 100 µL of a 3:7 methanol: water solution, after which the supernatant was collected and stored at −80 °C. Analysis of the supernatant was performed using HPLC‐MS/MS on a TripleTOF 7600 plus mass spectrometer (AB SCIEX, USA) coupled with an Agilent 1290 liquid chromatography system (Agilent, USA). For LC separation, the ACQUITY UPLC BEH C18 column (100 mm × 2.1 mm i.d., 1.7 µm; Waters) was utilized. The mobile phase consisted of A) formic acid in water (containing 25 mm ammonium acetate and 25 mm ammonia) and B) formic acid in acetonitrile (0:100, v/v). The flow rate was maintained at 0.5 mL min^−1^, with an injection volume of 2 µL. The column flow rate was fixed at 500 µL min^−1^, with the column temperature set to 40 °C. The chromatographic gradient was as follows: 0–0.5 min at 95% A; 0.5–7 min from 5% to 35% A; 7–8 min from 35% to 60% A; 8–9 min at 60% A; 9–9.1 min from 60% to 5% A; and 9.1–12 min at 5% A. Electrospray ionization MS was conducted in positive/negative ion modes. Information dependent acquisition (IDA) was employed to simultaneously gather full scan MS and MS/MS data. Mass data were collected within the m/z range of 60 to 1200 Da, with ion spray voltages set to 5000 V (positive mode) and 4000 V (negative mode), and a heated‐capillary temperature maintained at 600 °C. The flow rates for the curtain gas, nebulizer, and heater gas were established at 35, 60, and 60 arbitrary units, respectively, with the collision energy set to 30 V. The raw data generated were imported into Progenesis QI (version 2.2, Waters, USA) for peak detection, chromatogram deconvolution, alignment, and normalization. The coefficient of variance (CV) values for QC samples were calculated, and data with CVs less than 30% were preserved for further analysis. Subsequently, metabolites were identified by comparing the detected MS/MS spectra with information from the Human Metabolome Database (HMDB).

### Data Preprocessing

The mass spectrometry data preprocessing workflow included steps such as binning, smoothing, baseline correction, peak detection, and alignment. Spectrum smoothing was performed using a 1D Gaussian filter (sigma = 1) to eliminate noise. To reduce complexity, spectrum down‐sampling with a binning operation employing a window size of 0.05 Da was executed. Baselines were corrected using the white top‐hat operation through morphological transformations to eliminate background noise. Peak extraction and spectrum alignment were utilized to address inherent variations in mass‐to‐charge (m/z) ratios found across individual sample spectra. During peak extraction, raw spectral data from each sample were processed to identify and extract peaks using sophisticated algorithms aimed at distinguishing between signal and noise, ensuring high accuracy in the identified peaks for subsequent analysis. The spectrum alignment technique was utilized to standardize peak positions across all samples. In peak extraction, the raw spectral data from each sample was processed to identify and extract peaks, which involves sophisticated algorithms designed to differentiate between signal and noise, ensuring a high degree of accuracy in the peaks identified for subsequent analysis. In spectrum alignment, Spectrum Alignment technique was employed to standardize peak positions across all samples. This method meticulously adjusts the m/z positions to ensure that identical peaks across different spectra align perfectly. The primary goal of this alignment is to achieve consistency in the representation of each signal (identified peak) across the dataset, thereby enabling accurate cross‐sample comparisons. Local maxima operation was performed to extract the final metabolic signals for peak detection. As a result of these preprocessing efforts, the positions of 134 signals across 288 samples were successfully standardized, ensuring uniformity in m/z positions while acknowledging that signals intensities may vary among samples.

### Machine Learning

Machine learning of this work was conducted for model building, signal selection, parameter tuning, and validation on Python version 3.9 and Orange3‐3.37.0 (https://github.com/biolab/orange3). During model building, five machine learning algorithms, including Ridge Regression (RR), Adaptive Boosting (AdaBoost), K‐Nearest Neighbor (kNN), Naïve Bayes (NB), and Neural Network (NN) were used and trained using 10‐fold cross‐validation with 10 times repeating. The code for these five algorithms can be accessed through the following links: RR (https://github.com/biolab/orange3/blob/master/Orange/classification/logistic_regression.py), AdaBoost (https://github.com/biolab/orange3/blob/master/Orange/modelling/ada_boost.py), kNN (https://github.com/biolab/orange3/blob/master/Orange/modelling/knn.py), NB (https://github.com/biolab/orange3/blob/master/Orange/classification/naive_bayes.py), and NN (https://github.com/Alltnl/MMD). This data is available at the NIH Common Fund's National Metabolomics Data Repository (NMDR) website, the Metabolomics Workbench, https://www.metabolomicsworkbench.org where it has been assigned Project ID ST003368. The data can be accessed directly via it's Project DOI: 10.21228/M8B83W.

For the Neural Network (NN), the initial input was standardized to a 12D vector (x_input). This input comprised 134 signals for the recognition of serum metabolic fingerprints (SMFs) which included 134 mass‐to‐charge ratio (m/z) signals (x_spectral). To augment the networks' adaptability, the remaining features—10 for SMFs recognition—were assigned a value of 0. The architecture of these networks was rooted in deep neural networks (DNN), featuring two primary components: a feature extraction part (feature_extract) and a non‐linear feature interaction layer (feature_interaction). After undergoing extraction and interaction processes, the reorganized features were fed into a classification layer equipped with a Softmax function. This layer was responsible for generating the output probabilities for the classification tasks.^[^ ^36^ ^]^

For validation, all the machine learning algorithms were evaluated in an independent validation cohort.^[^ ^106^ ^]^ The stage prediction task was considered a binary classification. All included machine learning algorithms output a normalized probability of advanced stage from 0 to 1. For each machine learning model, the parameters are as follows: Ridge Regresstion: alpha = 0.140; Adaboost: base estimator = Tree, number of estimators = 60, learn rate = 1.0, classification algorithm = SAMME.R, regression loss function = Linear; kNN: number of neighbors = 5, weight = uniform, metric = Euclidean; naïve bayes: none; Neural Networks: batch_size_cv = 32, epochs_cv = 200, drop_rate = 0.25. During signal selection, the Integrated Gradient algorithm is used to illustrate the contribution of each signal in the NN algorithm, which is to obtain the contribution of non‐zero gradients in the unsaturated region to the importance of decisions by integrating gradients along different paths.^[^ ^83^ ^]^

### Power Analysis

Power analysis (FDR = 0.1, power = 0.9) was conducted on MetaboAnalyst 5.0 (https://www.metaboanalyst.ca/). The specifics of this method are as follows: Assume a set of test statistics measuring differential expression follows a normal distribution N (µ, σ^2^). Under the null hypothesis H_0_ (no differential expression), the mean µ = 0; under the alternative hypothesis H_1_ (differential expression), the mean µ ≠ 0. The cumulative distribution functions (CDF) of the test statistics under H_0_ and H_1_ are denoted as K and L, respectively. The observed test statistics' mixed CDF is given by:

(1) $$M\;\left(t\right)=\pi_{0}\;K\left(t\right)+\left(1-\pi_{0}\right)\int_{-\infty }^{+\infty }L\left(t,\;\theta \right)\lambda \left(\theta \right)d\theta$$

where λ represents the density of effect sizes θ, and π_0_ represents the proportion of non‐differentially expressed features.

(2) $$\int_{-\infty }^{+\infty }T\left(u,\;\theta \right)\lambda \left(\theta \right)d\theta =u\frac{\pi_{0}\left(1-\delta \right)}{\delta \left(1-\pi_{0}\right)}$$

The effect size is defined as the difference between the mean expression levels of metabolites under two conditions, divided by their pooled standard deviation. The estimation of π_0_ follows the approach suggested by Langaas et al.,^[^ ^107^ ^]^ and λ is estimated by a deconvolution estimator. The average power can be estimated by solving the following equation: where T represents the power for a single metabolite as a function of the p‐value u and effect size θ, and δ is the user‐defined false discovery rate. The average power is controlled for multiple testing through the adaptive Benjamini‐Hochberg method,^[^ ^108^ ^]^ which prevents overestimation. The effect size density estimation must be constrained to be non‐negative and integrated to 1. To avoid discontinuities where the constraint is applied, the π_0_ estimate needs to be readjusted. Estimating the average power also involves detecting effect sizes around zero, which are technically challenging to measure accurately. A small region around zero can be defined and excluded from the effect size density, thereby increasing the estimated average power.^[^ ^109^ ^]^

### Statistical Analysis

The heatmap, principal component analysis (PCA), and clustering analysis were performed on R version 4.2.2 using pheatmap, ggplot2, FactoMineR, factoextra, and NbClust packages.^[^ ^58^, ^110^ ^]^ Fold change and pathway analysis were conducted on MetaboAnalyst 5.0 (https://www.metaboanalyst.ca/).^[^ ^111^ ^]^ For pathway analysis, verification of the metabolites that displayed a significant difference between the HC group and MMD group was conducted on the human metabolome database (HMDB, http://www.hmdb.ca/) using the signal molecular formula.^[^ ^112^ ^]^ The pathway analysis was performed on MetaboAnalyst 5.0 based on the KEGG pathway library for homo sapiens. Another statistical analysis in this work was performed on SPSS software (version 24.0, SPSS Inc., USA) to calculate the P‐value for statistical demonstration, including two‐sided student's *t*‐test, chi‐square test, ANOVA, and Kruskal–Wallis H Test. All significance level was set as 0.05. AUC was calculated using Origin 2024.

## Conflict of Interest

The authors declare no conflict of interest.

## Author Contributions

Y.X., R.W., and X.G. contributed equally to this work. K.Q. and W.X. designed the overall approach and planned this work with Y.G. and W.N. R.W., X.G., J.S., M.Z., J.H. and H.Y. contributed to the serum sample collection and preparation, respectively. R.W. and X.G. also contributed to the summary of clinical information. Y.X., L.C., and W.X. contributed to the MS data acquisition and MS data analysis. Y.X. and W.X. organized the figure and wrote the manuscript with R.W. and X.G. All authors joined in the critical discussion and revised the manuscript. Additionally, we thank Tingting Xie for assisting the collection and treatment of blood samples.

## Supporting information



Supporting Information

## Data Availability

The data that support the findings of this study are available in the supplementary material of this article.
