# Supplementary material for: Non‐Invasive Diagnosis of Moyamoya Disease Using Serum Metabolic Fingerprints and Machine Learning
Source: Adv Sci (Weinh). 2024 Dec 31;12(8):2405580. doi: 10.1002/advs.202405580 (PMC11848555; doi:10.1002/advs.202405580)
Supplement: Supplementary file 1 — Supporting Information [file ADVS-12-2405580-s001.docx]

Supporting Information

Non-Invasive Diagnosis of Moyamoya Disease Using Serum Metabolic Fingerprints and Machine Learning

*Ruiyuan Weng ^†^, Yudian Xu ^†^,Xinjie Gao ^†^, Linlin Cao, Jiabin Su, Heng Yang, He Li, Chenhuan Ding, Jun Pu, Meng Zhang, Jiheng Hao, Wei Xu*,Wei Ni*, Kun Qian*, Yuxiang Gu**

**Supplementary Figures**


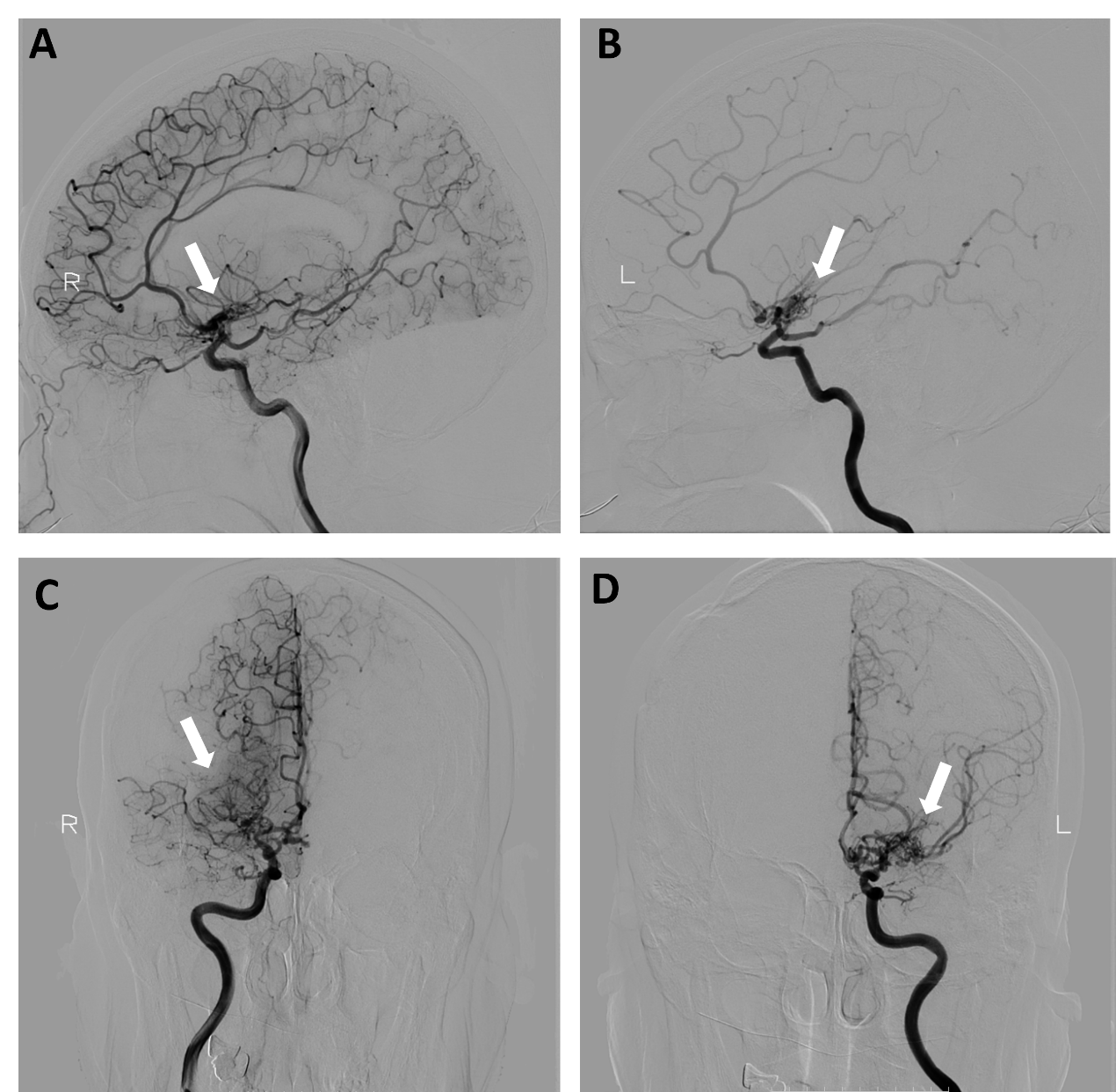


Figure S1. Typical Digital subtraction angiography (DSA) images of patients with MMD. Lateral DSA film of right (A) and left (B) internal carotid artery (ICA); post-anterior DSA film of right (C) and left (D) ICA. Arrows showed the abnormal small-vessels network (moyamoya vasculopathy).


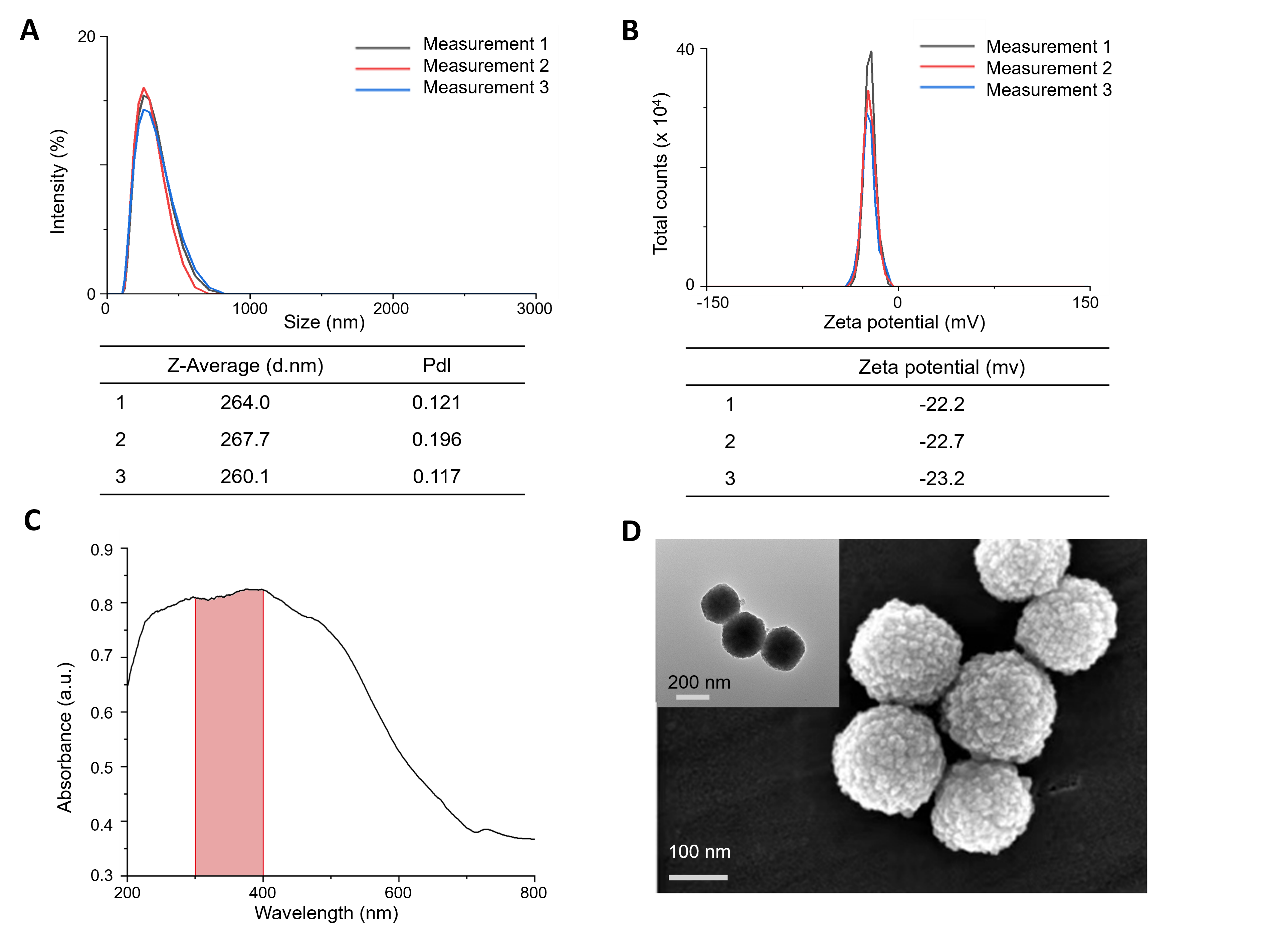


Figure S2. Characterization of the nanoparticles. Specifically, (A) Dynamic light scattering (DLS) and (B) zeta potential were recorded by 3 independent measurements. (C) Optical extinction spectrum of the nanoparticles. The red area at the wavelength of 300 to 400 nm indicated the efficient absorption and transfer of laser energy (Nd:YAG laser of 355 nm) in LDI MS. (D) Scanning Electron Microscope (SEM) image and Transmission Electron Microscope (TEM) image (inset) of the nanoparticles were displayed. The scale bar was 100 nm in d and 200 nm in the inset of d.


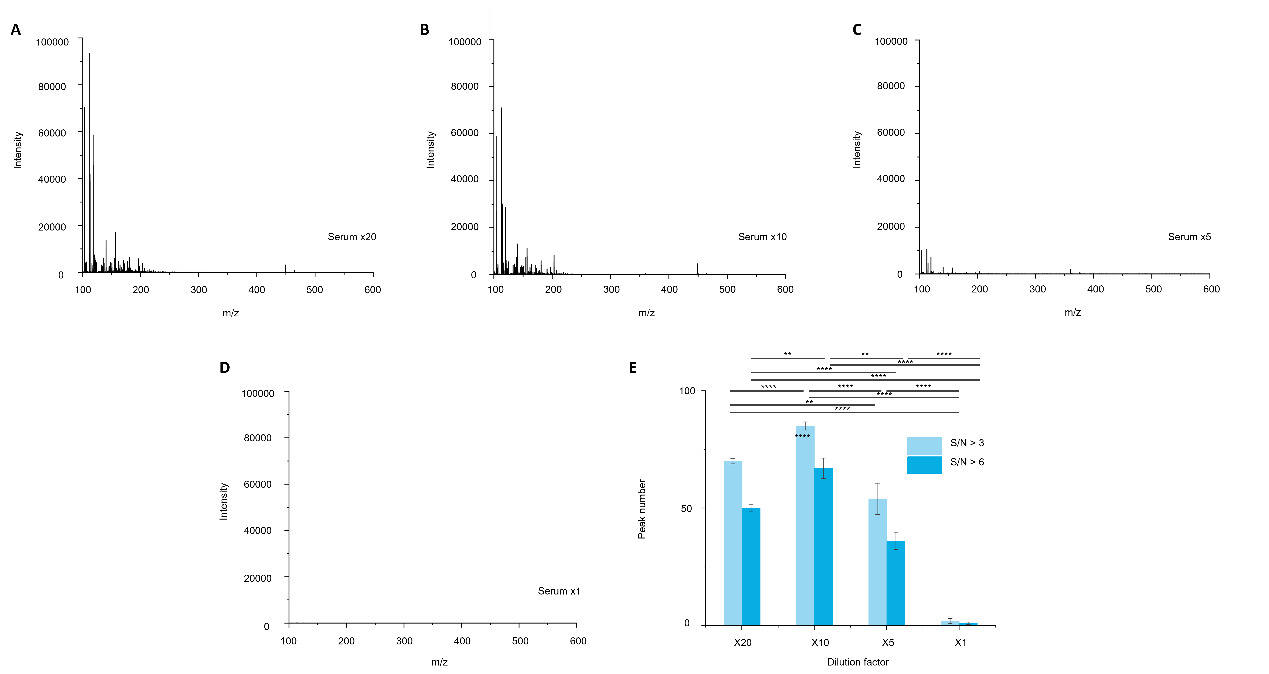


Figure S3. Optimization of serum dilution factor. The mass spectrum obtained by (B) diluting 10-fold serum were significantly better than that of (A) diluting 20-fold serum, (C) diluting 5-fold serum, and (D) original serum. (E) The number of peaks of S/N > 6 and S/N > 3 of the mass spectrum.


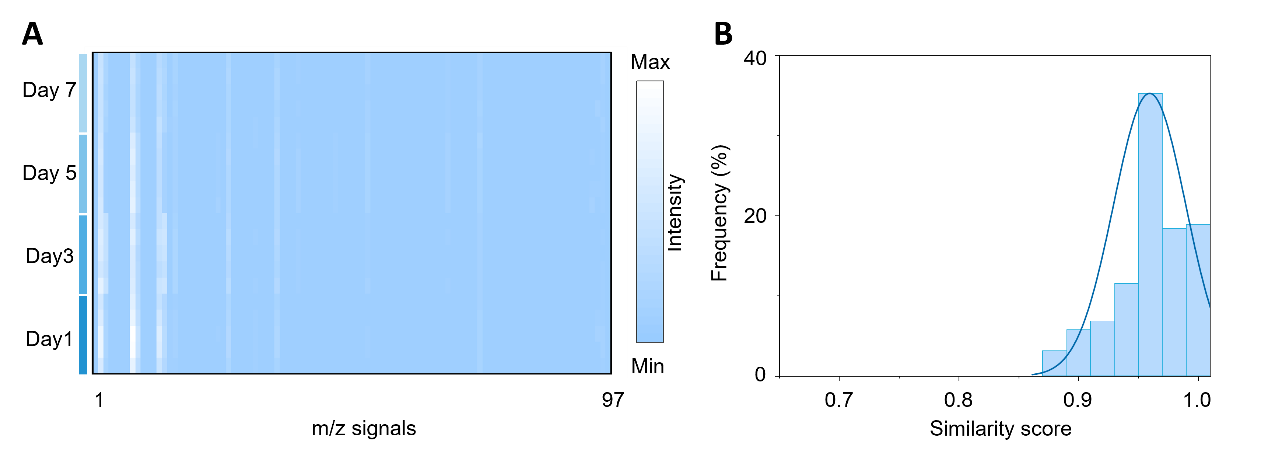


Figure S4. Platform stability detected in different time periods. (A) Heatmap of the metabolic fingerprint of standard serum on day 1, day 3, day 5, and day 7. (B) Similarity scores of the metabolic fingerprint of standard serum on day 1, day 3, day 5, and day 7.


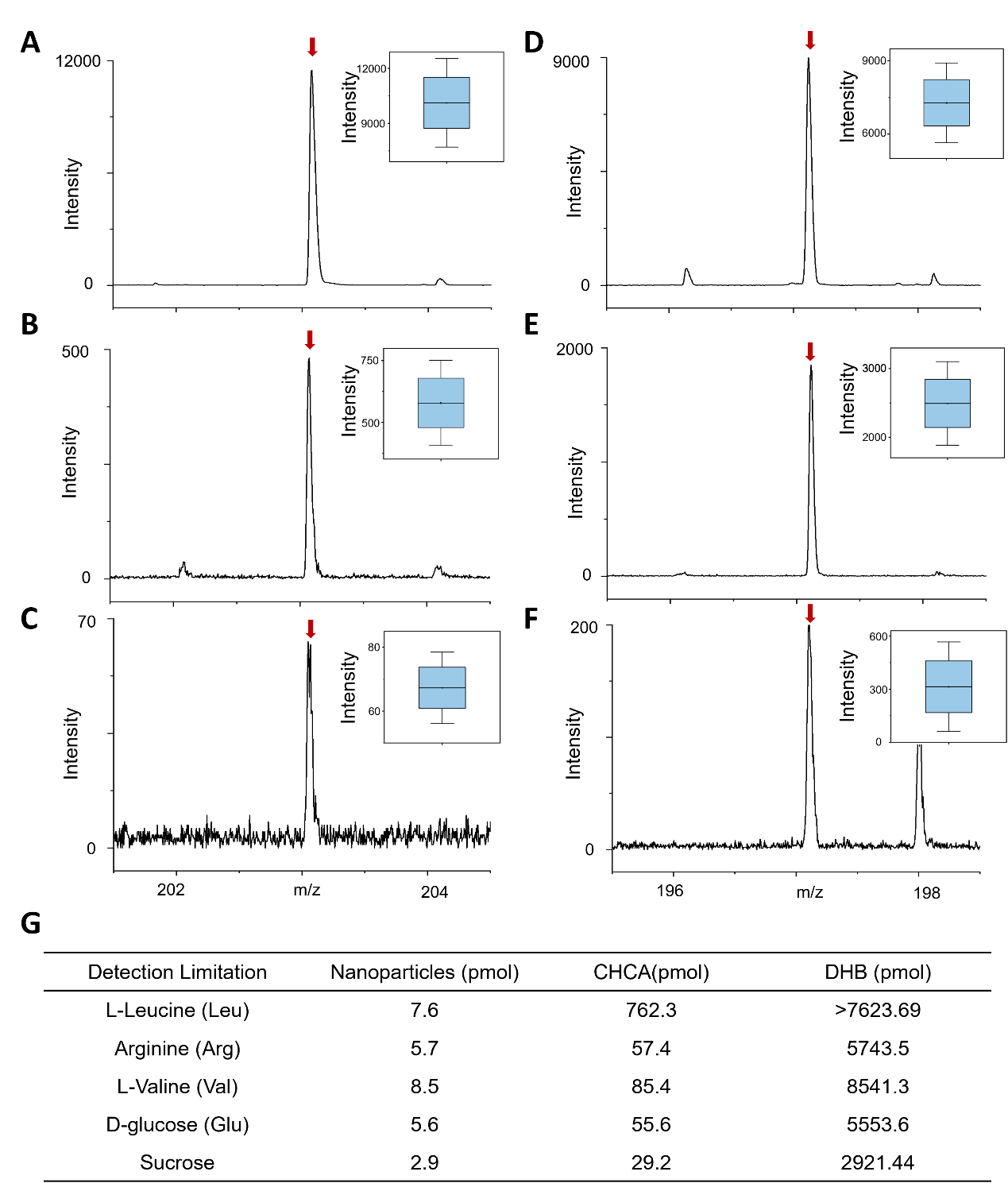


Figure S5. Detection limit using different matrices. Typical LDI MS using (A) nanoparticles, (B) alpha-cyano-4-hydroxycinnamic acid (CHCA), and (C) 2,5-Dihydroxybenzoic acid (DHB) were recorded with 55.6 pmol of Glu. Typical LDI MS using (D) nanoparticles, (E) CHCA, and (F) DHB were recorded with 57.4 pmol of Arg. The relevant m/z values of the analyte were marked with red arrows. (G) The summary of the detection limit for 5 standard small metabolites using nanoparticles, CHCA, and DHB.


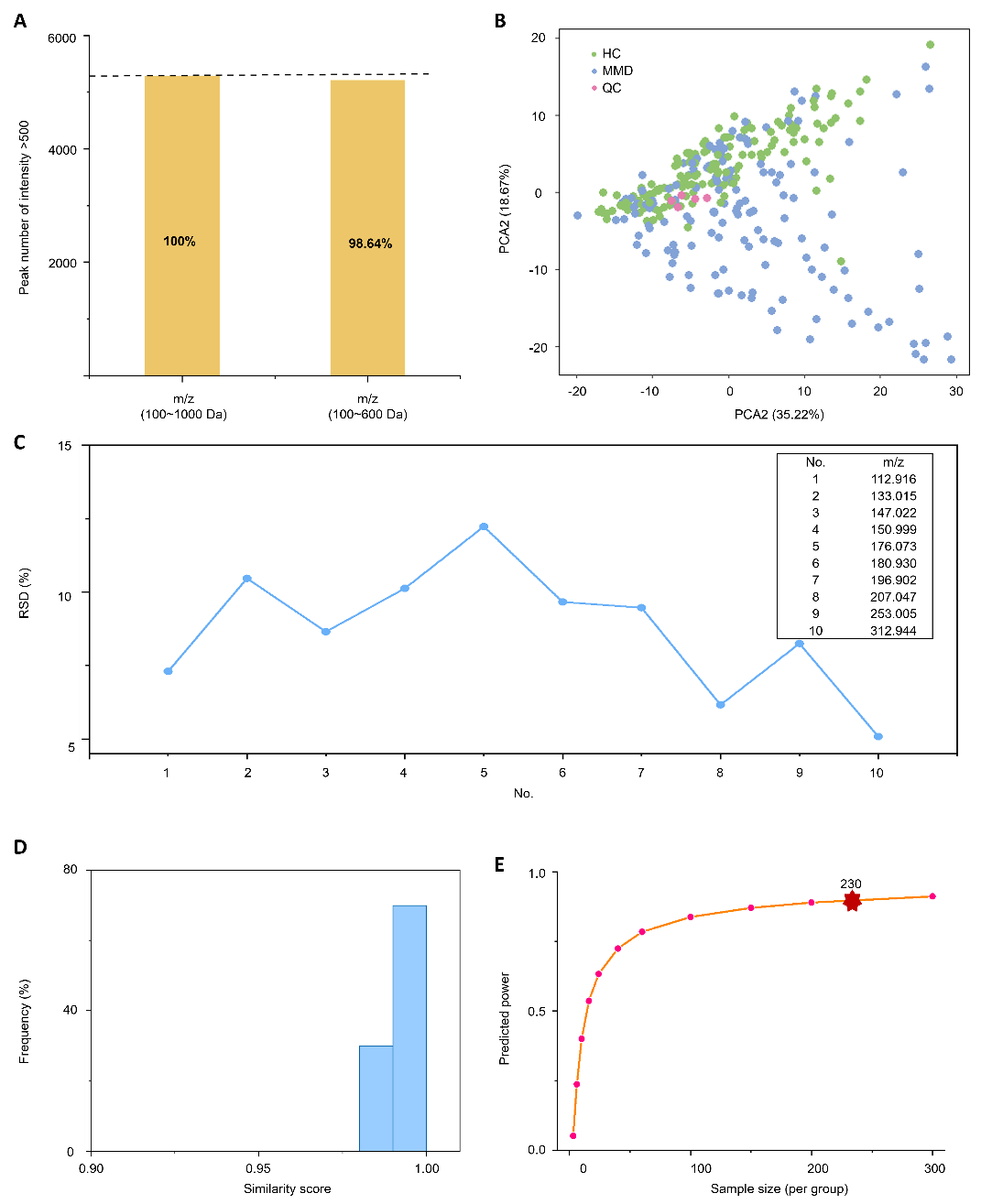


Figure S6. (A) The ratio of the peak number with intensity greater than 500 in the 100-600 Da interval to the peak number in the 100-1000 Da range. (B) PCA plot containing standard serum as quality control. (C) The relative standard deviation (RSD) of 10 m/z signals. (D) Similarity score of standard serum in real detection. (E) Power analysis based on SMF. The SMF of 10 samples (5/5, HC group/MMD group) was included to calculate the required sample number for machine learning.


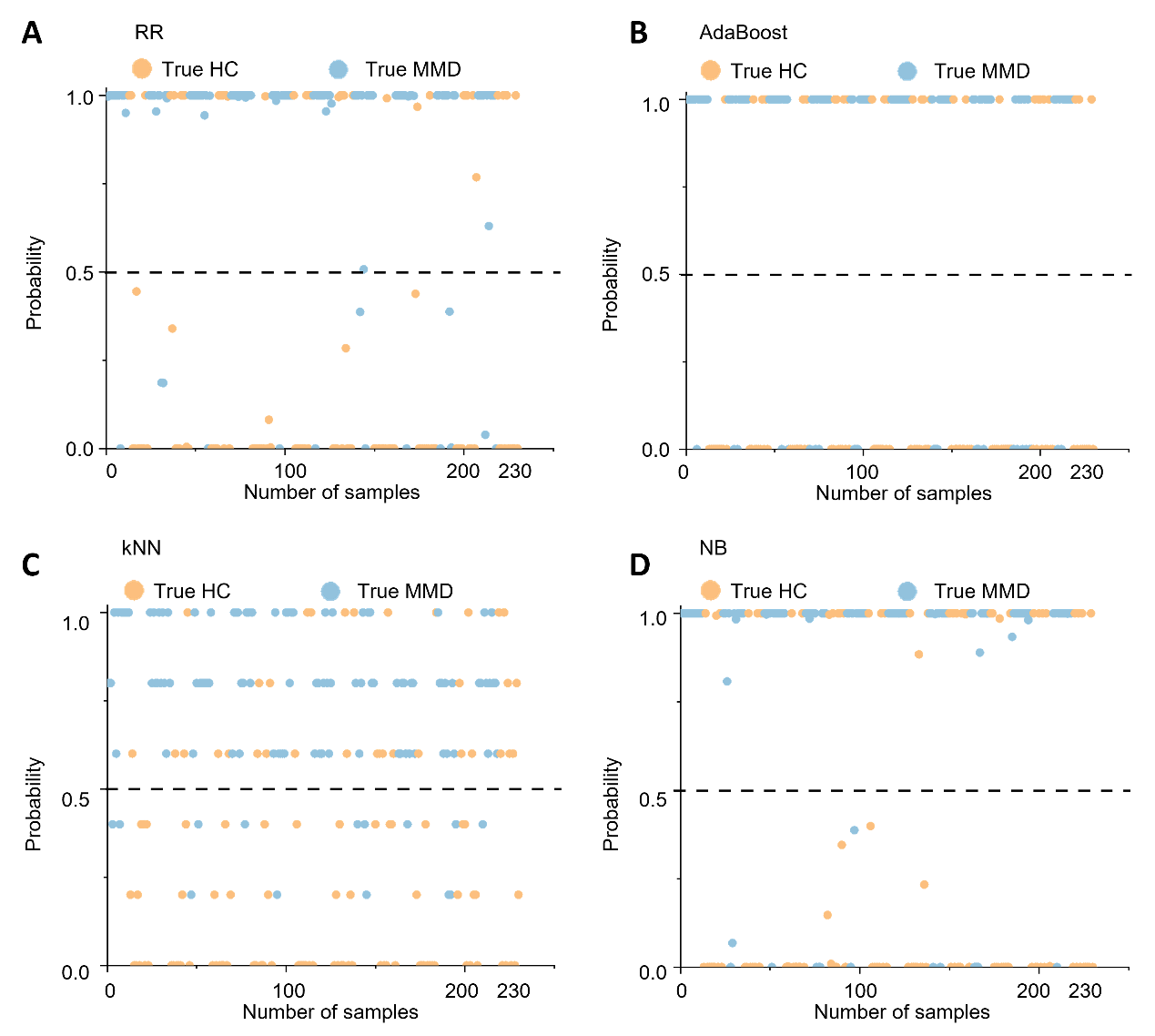


Figure S7. Diagnosis of MMD by different machine learning (ML) algorithms. A sample-level plot by (A) Ridge Regression (RR), (B) Adaptive Boosting (AdaBoost), (C) K-Nearest Neighbor (kNN), and (D) Naïve Bayes (NB), stratifying true HC (orange) and actual MMD patients (blue) for discovery cohort (n = 230; 144/144, HC / MMD group).


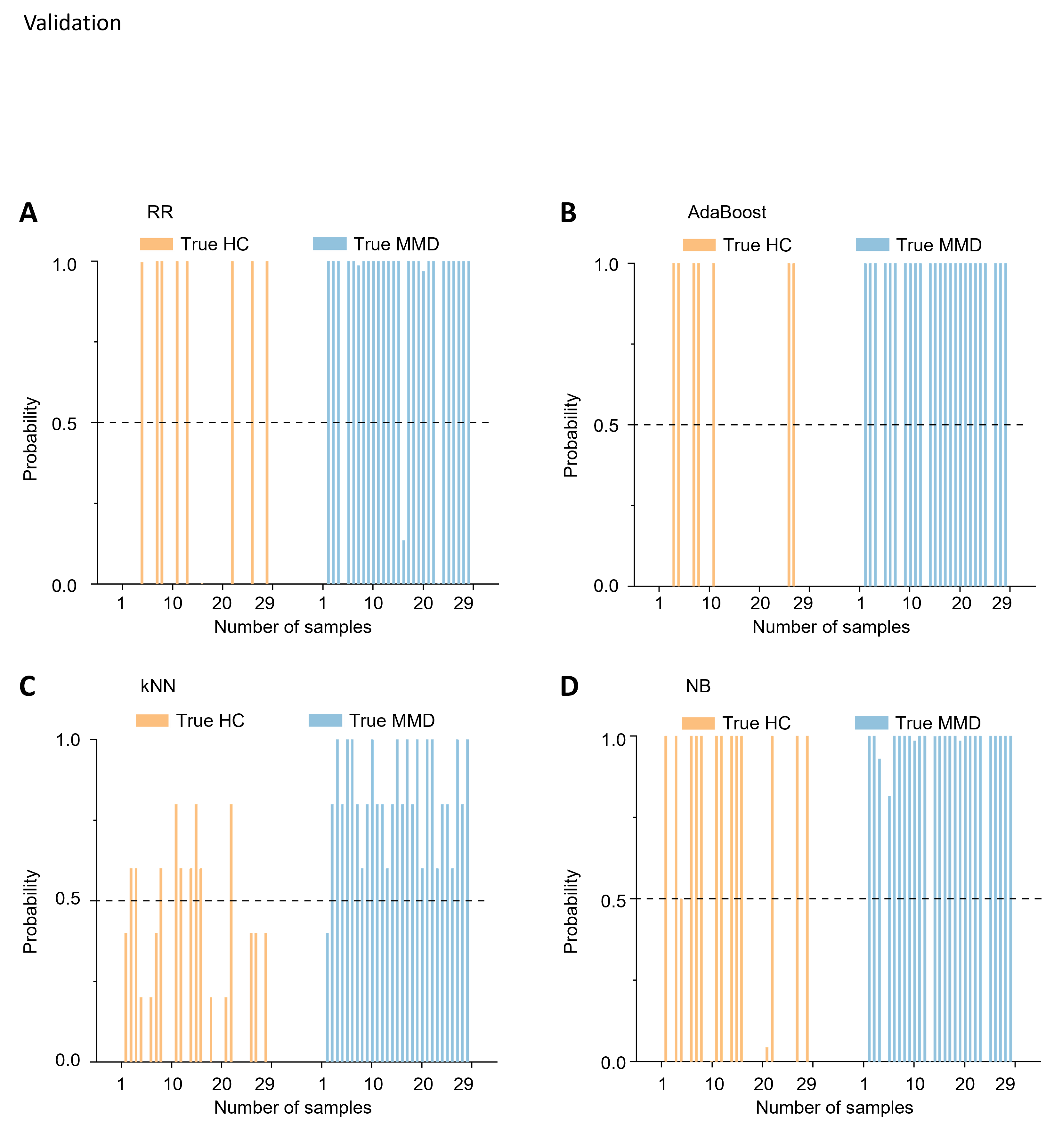


Figure S8. Diagnosis of MMD by different machine learning (ML) algorithms. A sample-level plot by (A) Ridge Regression (RR), (B) Adaptive Boosting (AdaBoost), (C) K-Nearest Neighbor (kNN), and (D) Naïve Bayes (NB), stratifying true HC (orange) and actual MMD patients (blue) for validation cohort (n = 58; 29/29, HC / MMD group).


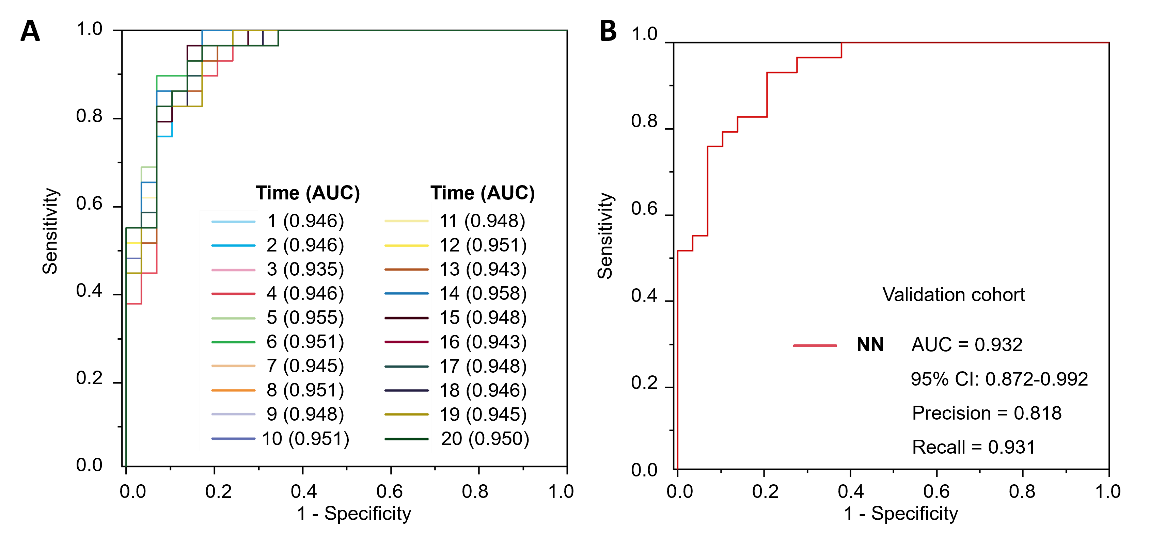


Figure S9. NN model stability verified through repeated validations. (A) The ROC curves for Neural Network algorithms (NN) for diagnosing the MMD group from the HC group in the validation cohort 20 times undergoing 10CV. (B) The ROC curve for Neural Network algorithms (NN) undergoing 20CV in the validation cohort.


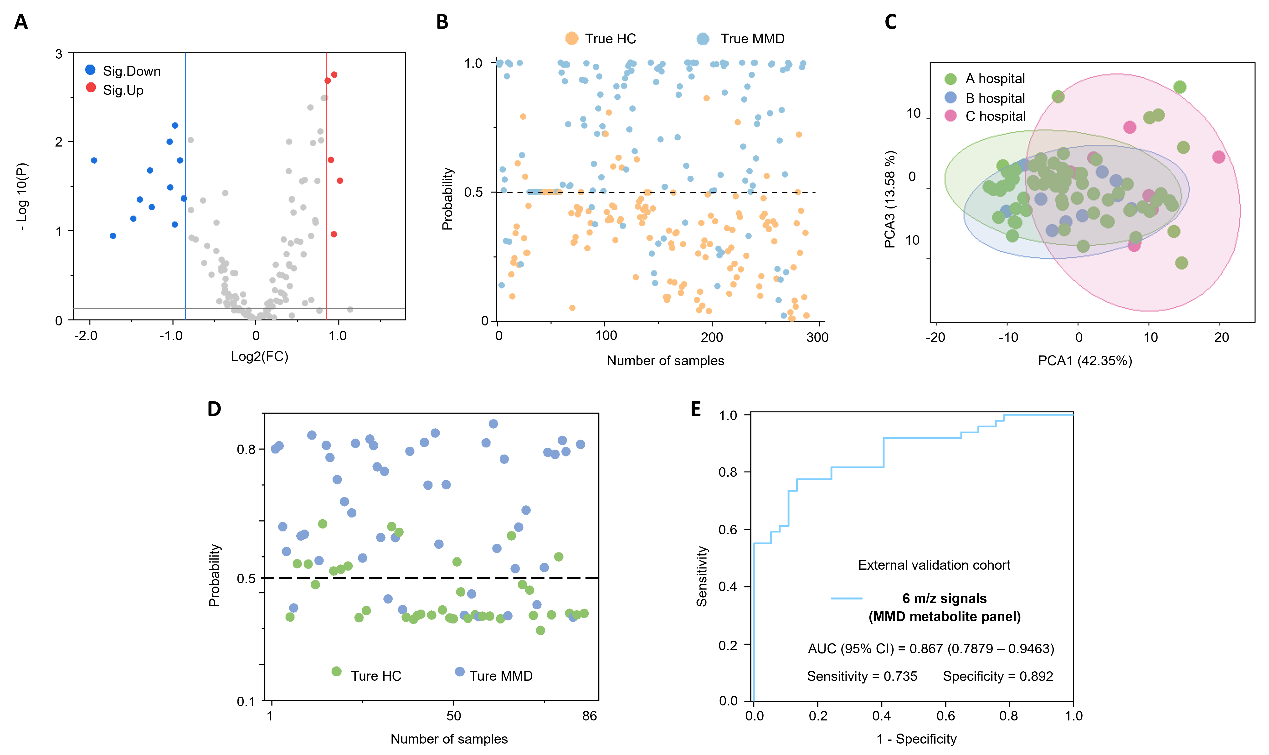


Figure S10. Signal selection. (A) 5 up-regulated signals and 12 down-regulated signals obtained by filtering the signals with |Log2(FC)| >1.8 and p <0.05. (B) A sample-level plot by NN algorithm, stratifying true HC (orange) and actual MMD patients (blue) for cohort (n = 288; 144/144, HC group / MMD group). (C) PCA plot of three different centers. (D) A sample-level plot by NN algorithm, stratifying true HC (green) and actual MMD patients (blue) for external validation cohort (n = 86; 37/49, HC group / MMD group). (E) The ROC curves for diagnosing the MMD group from the HC group in the external validation cohort.


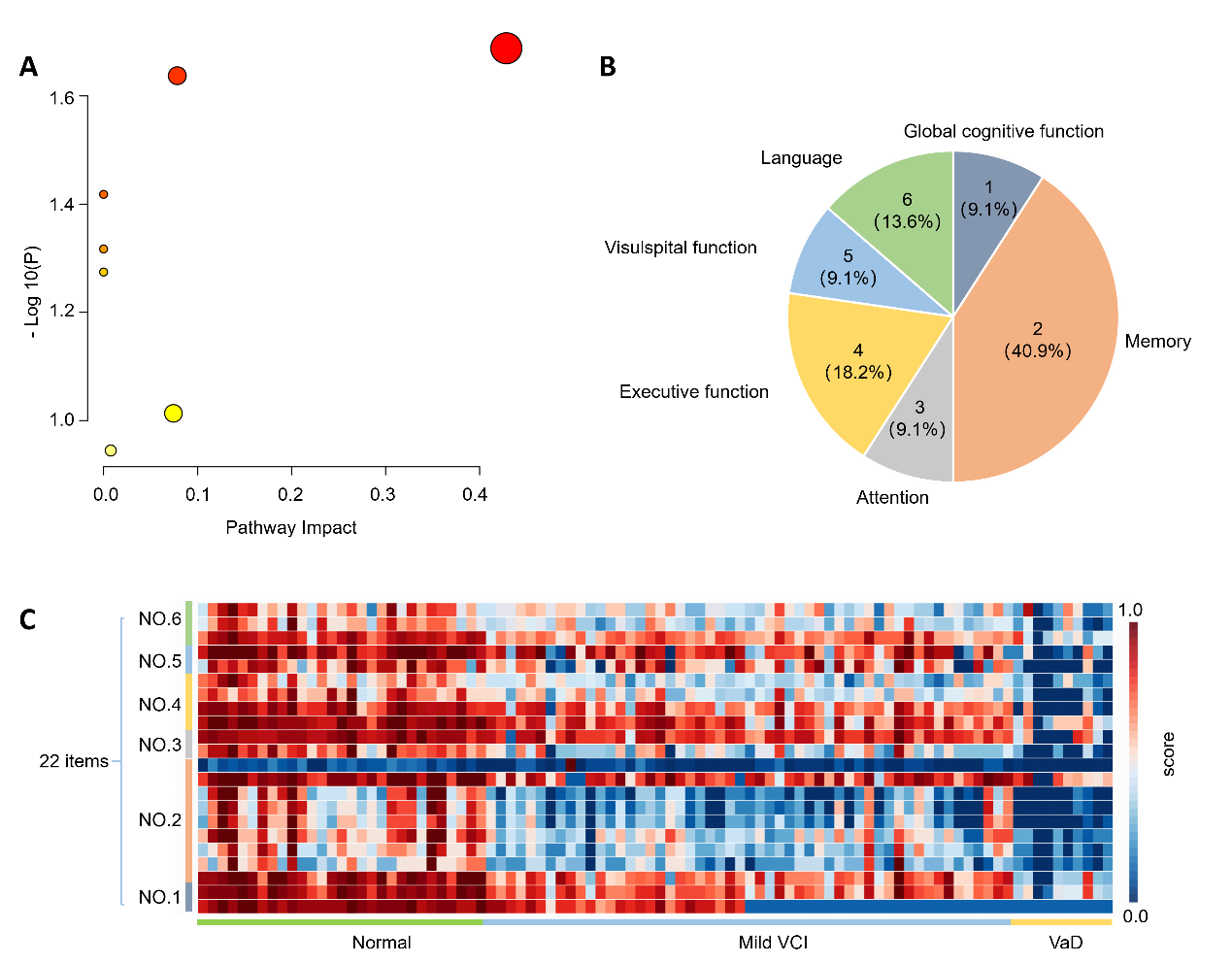


Figure S11. (A) Potential pathways differentially regulated in MMD group, compared to the HC group. The 6 selected metabolite signals were tested to identify altered pathways. The color and size of each circle were correlated to the P-value and pathway impact value. A total of seven pathways were differentially regulated: (1) aurine and hypotaurine metabolism, (2) vitamin B6 metabolism, (3) nicotinate and nicotinamide metabolism, (4) pantothenate and CoA biosynthesis, (5) beta-Alanine metabolism, (6) pyrimidine metabolism, and (7) primary bile acid biosynthesis. Three pathways (hypotaurine metabolism, vitamin B6 metabolism, and Pyrimidine metabolism) with an impact value greater than 0.05 are considered differentially altered between the MMD and HC groups. (B) The proportion of each of the six categories. (C) The distribution of cognitive impairment scores across 22 items in the normal, mild VCI group and VaD groups.


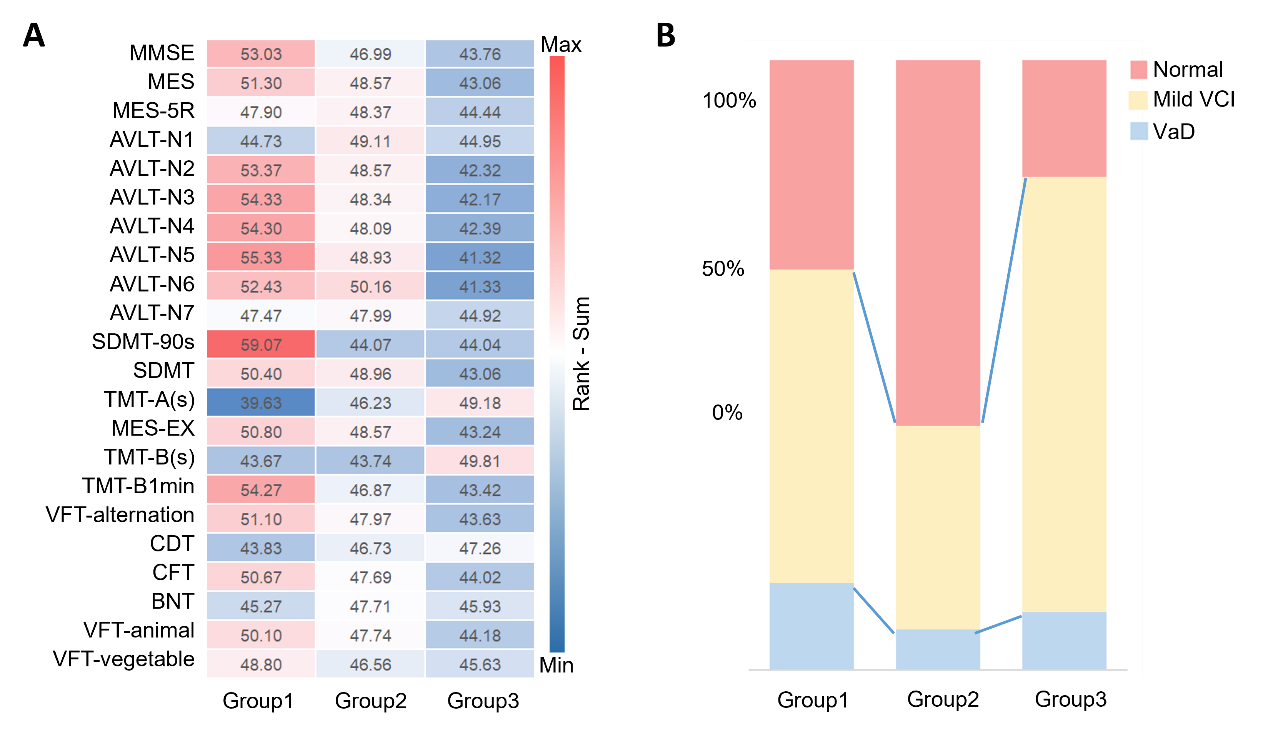


Figure S12. Association Between Cognitive Function and a MMD-Specific Panel of 6 Metabolites. (A) The heatmap of the rank-sum of neuropsychological scores indicates that Group 3 exhibited the poorest performance. (B) Group 3 comprised the highest proportion of mild Vascular Cognitive Impairment (VCI) and Vascular Dementia (VaD).

Table S1. Demographic information of 144 MMD and 144 HC group samples for

machine learning.

|  | MMD | Health Control | P |
| --- | --- | --- | --- |
| Age | 46.5±12.3 | 45.9±12.3 | 0.677^a)^ |
| Gender |  |  | 0.906^b)^ |
| Male | 67 (46.5%) | 68 (47.2%) |  |
| Female | 77 (53.5%) | 76 (52.8%) |  |
| BMI | 24.07±3.18 | 24.40±2.74 | 0.360 ^c)^ |
| Initial Clinical Presentation | |  |  |
| TIA/Infraction | 103 (71.5%) | - |  |
| Hemorrhage | 24 (16.7%) | - |  |
| Others | 17 (11.8) | - |  |
| Suzuki Stage |  |  |  |
| <IV | 79 (54.9%) | - |  |
| ≥IV | 65 (45.1%) | - |  |

a) The P-value was calculated by ANOVA.

b) The P-value was calculated by chi-square test.

c) The P-value was calculated by independent samples t-test.

Table S2. The performance of different machine learning algorithms as modeled by m/z signals.

| Algorithm | Discovery cohort | | | Validation cohort | | |
| --- | --- | --- | --- | --- | --- | --- |
|  | AUC  (95% CI) | Sen (%) | Spe (%) | AUC  (95% CI) | Sen (%) | Spe (%) |
| NN | 0.945  (0.912-0.977) | 0.991 | 0.887 | 0.958  (0.911-1.000) | 0.966 | 0.862 |
| RR | 0.824  (0.767-0.880) | 0.878 | 0.704 | 0.881  (0.793-0.968) | 0.897 | 0.724 |
| AdaBoost | 0.770  (0.707-0.833) | 0.783 | 0.757 | 0.810  (0.693-0.928) | 0.862 | 0.759 |
| kNN | 0.852  (0.801-0.903) | 0.878 | 0.704 | 0.929  (0.867-0.991) | 0.966 | 0.690 |
| NB | 0.789  (0.729-0.849) | 0.878 | 0.661 | 0.681  (0.539-0.823) | 0.897 | 0.552 |

NN: Neural Network; RR: Ridge Regression; AdaBoost: Adaptive Boosting; kNN: K-Nearest Neighbor; NB: Naïve Bayes.

Sen: Sensitivity, Sen = $\frac{TP}{TP + FN}$, TP= ture positives, FN = false negatives.

Spe: Specificity, Spe = $\frac{TN}{TN + FP}$, TN= ture negatives, FP = false positives.

Table S3. The DeLong test results of different algorithms compared with NN.

| Algorithm  vs NN | RR | AdaBoost | kNN | NB |
| --- | --- | --- | --- | --- |
| Discovery cohort | <0.001 | <0.0001 | 0.002 | <0.0001 |
| Validation cohort | 0.13 | <0.05 | 0.440 | <0.001 |

NN: Neural Network; RR: Ridge Regression; AdaBoost: Adaptive Boosting; kNN: K-Nearest Neighbor; NB: Naïve Bayes.

Table S4. The performance of different machine learning algorithms in five aspects: Accuracy, F1 score, Precision, Recall and MCC.

| Algorithm | Accuracy | F1 score | Precision | Recall | MCC |
| --- | --- | --- | --- | --- | --- |
| NN | 0.914 | 0.918 | 0.875 | 0.966 | 0.832 |
| RR | 0.810 | 0.825 | 0.765 | 0.897 | 0.630 |
| AdaBoost | 0.810 | 0.820 | 0.781 | 0.862 | 0.624 |
| kNN | 0.828 | 0.848 | 0.757 | 0.966 | 0.682 |
| NB | 0.724 | 0.765 | 0.667 | 0.897 | 0.478 |

NN: Neural Network; RR: Ridge Regression; AdaBoost: Adaptive Boosting; kNN: K-Nearest Neighbor; NB: Naïve Bayes.

MCC: Matthews Correlation Coefficient, MCC = $\frac{TP*TN-FP*FN}{\sqrt{(TP+FP)(TP+FN)(TN+FP)(TN+FN)}}$

Accuracy: Accuracy = $\frac{TP+TN}{TP+FP+TN+FN}$

F1 score: F1 score = $2*\frac{Precision*Recall}{Precision +Recall}$

Recall: Recall = $\frac{TP}{TP+FN}$

Precision: Precision = $\frac{TP}{TP+FP}$

TP= ture positives, FN = false negatives, TN= ture negatives, FP = false positives.

Table S5. The coefficient of variation of AUC value of 10 repeated instances for the five algorithms in the validation cohort.

| AUC | NN | RR | AdaBoost | kNN | NB |
| --- | --- | --- | --- | --- | --- |
| 1 | 0.958 | 0.828 | 0.759 | 0.858 | 0.694 |
| 2 | 0.952 | 0.856 | 0.828 | 0.917 | 0.839 |
| 3 | 0.955 | 0.951 | 0.879 | 0.876 | 0.845 |
| 4 | 0.955 | 0.870 | 0.690 | 0.922 | 0.761 |
| 5 | 0.948 | 0.895 | 0.707 | 0.867 | 0.700 |
| 6 | 0.950 | 0.901 | 0.690 | 0.911 | 0.810 |
| 7 | 0.949 | 0.910 | 0.707 | 0.821 | 0.764 |
| 8 | 0.947 | 0.911 | 0.741 | 0.791 | 0.816 |
| 9 | 0.950 | 0.845 | 0.759 | 0.913 | 0.841 |
| 10 | 0.955 | 0.834 | 0.862 | 0.945 | 0.925 |
| CV (%) | 0.37 | 4.31 | 8.81 | 5.26 | 8.40 |

NN: Neural Network; RR: Ridge Regression; AdaBoost: Adaptive Boosting; kNN: K-Nearest Neighbor; NB: Naïve Bayes.

Table S6. List of 134 potential metabolites with mass-to-charge ratios.

| m/z | HMDB ID | Molecular Formulas | Name | Ionization Mode | MWT (Da) |
| --- | --- | --- | --- | --- | --- |
| 100.962 | HMDB0245190 | C_2_H_6_OS | Beta-Mercaptoethanol | M+Na^+^ | 0.1604 |
| 103.061 | HMDB0 259828 | C_2_H_2_F_2_ | Vinylidene fluoride | M+K^+^ | 0.0720 |
| 103.979 | HMDB0244184 | C_3_H_3_N_3_ | 1,3,5-Triazine | M+Na^+^ | 0.0889 |
| 105.036 | HMDB0244222 | C_5_H_6_O | 1,4-Pentadien-3-one | M+Na^+^ | 0.0559 |
| 105.987 | HMDB0 243998 | C_5_H_9_N | 1-Piperideine | M+Na^+^ | 0.1364 |
| 106.990 | HMDB0004363 | C_3_H_4_N_2_O | Imidazolone | M+Na^+^ | 0.0766 |
| 108.995 | HMDB0256003 | C_3_H_2_O_3_ | Mesoxalaldehyde | M+Na^+^ | 0.0412 |
| 112.095 | HMDB0244944 | C_4_H_11_NO | 2-(Ethylamino)ethanol | M+Na^+^ | 0.0324 |
| 112.927 | HMDB0000190 | C_3_H_6_O_3_ | Lactic acid | M+Na^+^ | 0.1405 |
| 114.927 | HMDB0254464 | C_2_H_4_O_2_S | Mercaptoacetic acid | M+Na^+^ | 0.1796 |
| 116.934 | HMDB0254607 | CH_3_O_3_P | Methoxyphosphinate | M+Na^+^ | 0.0623 |
| 117.035 | HMDB0000228 | C_6_H_6_O | Phenol | M+Na^+^ | 0.1675 |
| 118.036 | HMDB0246752 | C_4_H_5_N_3_ | Pyrimidin-5-amine | M+Na^+^ | 0.0592 |
| 119.022 | HMDB0240714 | CH_5_O_3_P | Methylphosphonic acid | M+Na^+^ | 0.0095 |
| 119.959 | HMDB0034830 | H_3_NO_3_S | Amidosulfonic acid | M+Na^+^ | 0.1252 |
| 121.010 | HMDB0001429 | H_3_O_4_P | Phosphate | M+Na^+^ | 0.0250 |
| 121.962 | HMDB0259155 | C_2_HN_3_O_2_ | Triazolinedione | M+Na^+^ | 0.0764 |
| 122.965 | HMDB0250981 | C_5_H_8_O_2_ | Delta-Valerolactone | M+Na^+^ | 0.1420 |
| 124.978 | HMDB0244035 | C_2_H_2_F_4_ | 1,1,1,2-Tetrafluoroethane | M+Na^+^ | 0.0438 |
| 128.074 | HMDB0244975 | C_4_H_11_NO_2_ | 2-Amino-2-methyl-1,3-propanediol | M+Na^+^ | 0.0516 |
| 129.050 | HMDB0000139 | C_3_H_6_O_4_ | Glyceric acid | M+Na^+^ | 0.0173 |
| 129.978 | HMDB0257419 | C_2_H_5_NO_2_S | S-Nitrosomercaptoethanol | M+Na^+^ | 0.1417 |
| 131.053 | HMDB0001858 | C_7_H_8_O | P-Cresol | M+Na^+^ | 0.0749 |
| 133.027 | HMDB0000957 | C_6_H_6_O_2_ | Pyrocatechol | M+Na^+^ | 0.0735 |
| 134.062 | HMDB0000630 | C_4_H_5_N_3_O | Cytosine | M+Na^+^ | 0.0300 |
| 135.044 | HMDB0032233 | C_6_H_8_O_2_ | 2,5-Dimethyl-3(2H)-furanone | M+Na^+^ | 0.0720 |
| 136.099 | HMDB0256489 | H_4_NO_3_P | Phosphoramidic acid | M+K^+^ | 0.0412 |
| 136.979 | HMDB0258046 | C_2_HF_3_O_2_ | Fluoro 2,2-difluoroacetate | M+Na^+^ | 0.0334 |
| 138.107 | HMDB0000162 | C_5_H_9_NO_2_ | L-Proline | M+Na^+^ | 0.0132 |
| 138.949 | HMDB0000134 | C_4_H_4_O_4_ | Fumaric acid | M+Na^+^ | 0.1133 |
| 140.122 | HMDB0245176 | C_4_H_7_NS | 2-Iminothiolane | M+K^+^ | 0.1468 |
| 140.949 | HMDB0000202 | C_4_H_6_O_4_ | Methylmalonic acid | M+Na^+^ | 0.1291 |
| 142.097 | HMDB0246586 | C_3_H_5_NOS | 4-Thiazolidinone | M+K^+^ | 0.1415 |
| 142.963 | HMDB0251494 | C_2_H_4_N_2_S_2_ | Dithiooxamide | M+Na^+^ | 0.2168 |
| 144.053 | HMDB0000574 | C_3_H_7_NO_2_S | L-Cysteine | M+Na^+^ | 0.0952 |
| 145.029 | HMDB0001406 | C_6_H_6_N_2_O | Niacinamide | M+Na^+^ | 0.0851 |
| 145.967 | HMDB0001488 | C_6_H_5_NO_2_ | Nicotinic acid | M+Na^+^ | 0.1319 |
| 147.035 | HMDB0252053 | C_3_H_8_O_3_S | Ethyl methanesulfonate | M+Na^+^ | 0.1051 |
| 147.975 | HMDB0000251 | C_2_H_7_NO_3_S | Taurine | M+Na^+^ | 0.1618 |
| 148.999 | HMDB0000262 | C_5_H_6_N_2_O_2_ | Thymine | M+Na^+^ | 0.1043 |
| 150.039 | HMDB0060659 | C_4_H_5_N_3_O_2_ | 1-Methyl-4-nitroimidazole | M+Na^+^ | 0.0523 |
| 151.019 | HMDB0000300 | C_4_H_4_N_2_O_2_ | Uracil | M+K^+^ | 0.1664 |
| 152.070 | HMDB0000070 | C_6_H_11_NO_2_ | Pipecolic acid | M+Na^+^ | 0.0767 |
| 152.988 | HMDB0246829 | C_3_H_2_N_2_O_2_S | 5-Nitrothiazole | M+Na^+^ | 0.1220 |
| 154.132 | HMDB0000162 | C_5_H_9_NO_2_ | L-Proline | M+K^+^ | 0.0965 |
| 154.918 | HMDB0000223 | C_4_H_4_O_5_ | Oxalacetic acid | M+Na^+^ | 0.1435 |
| 156.091 | HMDB0245453 | C_3_H_3_NO_2_S | 2,4-Dihydroxythiazole | M+K^+^ | 0.1270 |
| 156.921 | HMDB0000156 | C_4_H_6_O_5_ | Malic acid | M+Na^+^ | 0.1562 |
| 158.076 | HMDB0246353 | C_7_H_9_N_3_ | 4-Aminobenzamidine | M+Na^+^ | 0.0840 |
| 158.933 | HMDB0000157 | C_5_H_4_N_4_O | Hypoxanthine | M+Na^+^ | 0.1687 |
| 160.086 | HMDB0000306 | C_8_H_11_NO | Tyramine | M+Na^+^ | 0.0823 |
| 161.006 | HMDB0246780 | C_4_H_2_N_4_O_2_ | 5-Diazouracil | M+Na^+^ | 0.0702 |
| 161.946 | HMDB0245317 | C_2_H_5_NO_4_S | 2-Sulfamoylacetic Acid | M+Na^+^ | 0.1743 |
| 162.100 | HMDB0 001232 | C_6_H_5_NO_3_ | 4-Nitrophenol | M+Na^+^ | 0.0016 |
| 163.008 | HMDB0002271 | C_6_H_8_N_2_O_2_ | Imidazolepropionic acid | M+Na^+^ | 0.1219 |
| 163.945 | HMDB0256486 | CH_4_NO_5_P | Phosphonoaminocarboxylic acid | M+Na^+^ | 0.0641 |
| 164.973 | HMDB0061735 | C_2_H_7_O_3_PS | Dimethylthiophosphate | M+Na^+^ | 0.1304 |
| 166.023 | HMDB0004827 | C_7_H_13_NO_2_ | Proline betaine | M+Na^+^ | 0.1501 |
| 166.991 | HMDB0000079 | C_5_H_8_N_2_O_2_ | Dihydrothymine | M+K^+^ | 0.1398 |
| 168.016 | HMDB0247069 | C_4_H_4_FN_3_O_2_ | 6-Hydroxy-5-fluorocytosine | M+Na^+^ | 0.0668 |
| 169.003 | HMDB0248341 | C_4_H_6_N_2_O_4_ | Ammonia aspartate | M+Na^+^ | 0.0885 |
| 169.112 | HMDB0245615 | C_4_H_6_N_2_O_2_S | 2-Amino-2-thiazoline-4-carboxylic acid | M+Na^+^ | 0.0382 |
| 170.089 | HMDB0243920 | C_10_H_13_N | 1-Methyl-1,2,3,4-tetrahydroisoquinoline | M+Na^+^ | 0.1222 |
| 170.896 | HMDB0000426 | C_5_H_8_O_5_ | Citramalic acid | M+Na^+^ | 0.2080 |
| 172.895 | HMDB0000283 | C_5_H_10_O_5_ | D-Ribose | M+Na^+^ | 0.2250 |
| 174.057 | HMDB0001859 | C_8_H_9_NO_2_ | Acetaminophen | M+Na^+^ | 0.0958 |
| 174.905 | HMDB0000786 | C_5_H_4_N_4_O_2_ | Oxypurinol | M+Na^+^ | 0.1954 |
| 175.048 | HMDB0000292 | C_5_H_4_N_4_O_2_ | Xanthine | M+Na^+^ | 0.0529 |
| 176.106 | HMDB0000073 | C_8_H_11_NO_2_ | Dopamine | M+Na^+^ | 0.0619 |
| 177.922 | HMDB0258990 | C_6_H_5_NS_2_ | Thienothiazine | M+Na^+^ | 0.1446 |
| 178.075 | HMDB0254533 | C_8_H_13_NS | Methiopropamine | M+Na^+^ | 0.1746 |
| 178.985 | HMDB0000226 | C_5_H_4_N_2_O_4_ | Orotic acid | M+Na^+^ | 0.1013 |
| 179.925 | HMDB0256486 | CH_4_NO_5_P | Phosphonoaminocarboxylic acid | M+K^+^ | 0.1926 |
| 180.055 | HMDB0000224 | C_2_H_8_NO_4_P | O-Phosphoethanolamine | M+K^+^ | 0.1061 |
| 180.947 | HMDB0061737 | C_2_H_7_O_2_PS_2_ | Dimethyldithiophosphate | M+Na^+^ | 0.2233 |
| 182.009 | HMDB0258541 | C_5_H_5_NO_5_ | Succinimidyl carbonate | M+Na^+^ | 0.0781 |
| 182.942 | HMDB0000225 | C_6_H_8_O_5_ | Oxoadipic acid | M+Na^+^ | 0.1721 |
| 184.119 | HMDB0006078 | C_7_H_17_N_2_O_2_ | Putreanine | M+Na^+^ | 0.0930 |
| 185.082 | HMDB0 000641 | C_5_H_10_N_2_O_3_ | L-Glutamine | M+K^+^ | 0.1612 |
| 186.009 | HMDB0001890 | C_5_H_9_NO_3_S | Acetylcysteine | M+Na^+^ | 0.1756 |
| 186.967 | HMDB0256333 | C_3_HF_5_O_2_ | Pentafluoropropionic acid | M+Na^+^ | 0.0536 |
| 188.114 | HMDB0 000897 | C_6_H_7_N_5_O | 7-Methylguanine | M+Na^+^ | 0.0289 |
| 188.982 | HMDB0001886 | C_6_H_6_N_4_O_2_ | 3-Methylxanthine | M+Na^+^ | 0.1454 |
| 189.967 | HMDB0 000232 | C_7_H_5_NO_4_ | Quinolinic acid | M+Na^+^ | 0.1419 |
| 191.089 | HMDB0251459 | C_13_H_12_ | Diphenylmethane | M+Na^+^ | 0.1399 |
| 192.079 | HMDB0245011 | C_12_H_11_N | 2-Aminobiphenyl | M+Na^+^ | 0.1374 |
| 193.044 | HMDB0014690 | C_7_H_10_N_2_OS | Propylthiouracil | M+Na^+^ | 0.1783 |
| 194.049 | HMDB0015052 | C_6_H_9_N_3_O_3_ | Metronidazole | M+Na^+^ | 0.0950 |
| 194.945 | HMDB0255875 | C_3_H_9_O_2_PS_2_ | O,S,S-Trimethyl phosphorodithioate | M+Na^+^ | 0.2447 |
| 196.033 | HMDB0248767 | C_5_H_7_N_3_O_4_ | Azaserine | M+Na^+^ | 0.0847 |
| 196.919 | HMDB0256424 | C_6_H_7_O_2_PS | Phenyl phosphonothioate | M+Na^+^ | 0.2204 |
| 198.135 | HMDB0 000904 | C_6_H_13_N_3_O_3_ | Citrulline | M+Na^+^ | 0.0403 |
| 198.918 | HMDB0000044 | C_6_H_8_O_6_ | Ascorbic acid | M+Na^+^ | 0.1955 |
| 200.091 | HMDB0001855 | C_10_H_11_NO_2_ | 5-Hydroxytryptophol | M+Na^+^ | 0.0229 |
| 200.977 | HMDB0 000150 | C_6_H_10_O_6_ | Gluconolactone | M+Na^+^ | 0.1530 |
| 201.983 | HMDB0001514 | C_6_H_13_NO_5_ | Glucosamine | M+Na^+^ | 0.1781 |
| 202.937 | HMDB0246988 | C_4_H_2_F_6_O | Fluoromethyl 2,2-difluoro-1-(trifluoromethyl)vinyl ether | M+Na^+^ | 0.1019 |
| 203.100 | HMDB0000122 | C_6_H_12_O_6_ | D-Glucose | M+Na^+^ | 0.0455 |
| 204.112 | HMDB0248794 | C_9_H_15_N_3_O | Azepexole | M+Na^+^ | 0.1172 |
| 205.016 | HMDB0245171 | C_8_H_6_O_5_ | 2-Hydroxyterephthalic acid | M+Na^+^ | 0.1048 |
| 205.942 | HMDB0 000017 | C_8_H_9_NO_4_ | 4-Pyridoxic acid | M+Na^+^ | 0.2088 |
| 207.066 | HMDB0000263 | C_3_H_5_O_6_P | Phosphoenolpyruvic acid | M+K^+^ | 0.0745 |
| 208.062 | HMDB0000239 | C_8_H_11_NO_3_ | Pyridoxine | M+K^+^ | 0.1159 |
| 209.066 | HMDB0243803 | C_11_H_10_N_2_O | 1-Naphthylurea | M+Na^+^ | 0.1383 |
| 210.102 | HMDB0001276 | C_9_H_21_N_3_O | N1-Acetylspermidine | M+Na^+^ | 0.1706 |
| 210.989 | HMDB0000670 | C_7_H_16_N_4_O_2_ | Homo-L-arginine | M+Na^+^ | 0.2283 |
| 214.099 | HMDB0006468 | C_10_H_13_N_3_O | 4-Hydroxydebrisoquine | M+Na^+^ | 0.1202 |
| 214.893 | HMDB0000193 | C_6_H_8_O_7_ | Isocitric acid | M+Na^+^ | 0.2201 |
| 216.066 | HMDB0000821 | C_10_H_11_NO_3_ | Phenylacetylglycine | M+Na^+^ | 0.1226 |
| 216.890 | HMDB0000250 | H_4_O_7_P_2_ | Pyrophosphate | M+Na^+^ | 0.1839 |
| 220.105 | HMDB0246347 | C_14_H_15_N | 2,3-Dimethyl-4-aminobiphenyl | M+Na^+^ | 0.1659 |
| 220.987 | HMDB0004063 | C_10_H_15_NO_3_ | Metanephrine | M+Na^+^ | 0.1159 |
| 221.918 | HMDB0011185 | C_4_H_10_NO_6_P | O-Phosphothreonine | M+Na^+^ | 0.1707 |
| 223.038 | HMDB0001866 | C_8_H_8_O_5_ | 3,4-Dihydroxymandelic acid | M+K^+^ | 0.2066 |
| 225.046 | HMDB0240258 | C_8_H_10_O_6_ | Succinylacetoacetate | M+Na^+^ | 0.0087 |
| 226.084 | HMDB0002096 | C_12_H_13_NO_2_ | 3-Indolebutyric acid | M+Na^+^ | 0.1427 |
| 226.947 | HMDB0244212 | C_11_H_8_O_4_ | 1,4-Dihydroxy-2-naphthoic acid | M+Na^+^ | 0.2214 |
| 228.983 | HMDB0245962 | C_6_H_6_O_8_ | 3-Oxalomalic acid | M+Na^+^ | 0.1137 |
| 232.043 | HMDB0248757 | C_13_H_7_NO_2_ | Aza-anthracenedione | M+Na^+^ | 0.1504 |
| 234.964 | HMDB0248942 | C_11_H_14_ClN | Benalfocin | M+K^+^ | 0.1760 |
| 235.108 | HMDB0014382 | C_10_H_16_N_2_O_3_ | Butabarbital | M+Na^+^ | 0.1273 |
| 236.090 | HMDB0253757 | C_10_H_15_NO_4_ | Kainic acid | M+Na^+^ | 0.1331 |
| 237.052 | HMDB0240715 | C_13_H_10_O_3_ | 2,4-Dihydroxybenzophenone | M+Na^+^ | 0.1579 |
| 238.934 | HMDB0014470 | C_5_FeN_6_O | Nitroprusside | M+Na^+^ | 0.0061 |
| 242.066 | HMDB0255868 | C_8_H_13_NO_6_ | O-Succinyl-L-homoserine | M+Na^+^ | 0.1162 |
| 242.915 | HMDB0246477 | C_6_H_5_IO | 4-Iodophenol | M+Na^+^ | 0.0824 |
| 249.119 | HMDB0253672 | C_11_H_18_N_2_O_3_ | Isopilocarpinic acid | M+Na^+^ | 0.1466 |
| 250.944 | HMDB0246482 | C_6_H_4_N_4_O_4_S | 4-Ketoniridazole | M+Na^+^ | 0.2258 |
| 253.024 | HMDB0000618 | C_5_H_11_O_8_P | D-Ribulose 5-phosphate | M+Na^+^ | 0.0754 |
| 254.902 | HMDB0250384 | C_9_H_4_N_4_O_4_ | 6-Cyano-7-nitroquinoxaline-2,3-dione | M+Na^+^ | 0.2427 |
| 265.086 | HMDB0 254199 | C_12_H_10_N_4_O_2_ | Lumichrome | M+Na^+^ | 0.1370 |
| 268.995 | HMDB0 015153 | C_9_H_9_Cl_2_N_3_O | Guanfacine | M+Na^+^ | 0.0881 |
| 275.004 | HMDB0 000071 | C_10_H_12_N_4_O_4_ | Deoxyinosine | M+Na^+^ | 0.2163 |
| 290.981 | HMDB0 000195 | C_10_H_12_N_4_O_5_ | Inosine | M+Na^+^ | 0.2349 |

MWD: Molecular Weight Deviation (|MWD _original molecular_ + MWD _Na/K_^+^ - MWD _query m/z_|)

Table S7. The HMDB ID of the potential biomarkers for MMD diagnosis.

| m/z  (LDI MS） | HMDB ID | Name | MWD (ppm) | m/z (FT ICR) | MWD (ppm) |
| --- | --- | --- | --- | --- | --- |
| 145.967 | HMDB0001488 | Nicotinic acid | 369 | 146.024 | 19 |
| 147.975 | HMDB0000251 | Taurine | 195 | 147.982 | 148 |
| 151.019 | HMDB0000300 | Uracil | 187 | 150.982 | 56 |
| 166.991 | HMDB0000079 | Dihydrothymine | 183 | 167.025 | 20 |
| 208.062 | HMDB0000239 | Pyridoxine | 119 | 208.049 | 60 |
| 225.046 | HMDB0240258 | Succinylacetoacetate | 38 | 225.044 | 33 |

MWD: Molecular Weight Deviation (|(query m/z – molecule adduct m/z)/molecule adduct m/z|*1000000)

Table S8. The performance of metabolites panel using NN algorithm.

| m/z | AUC  (95% CI) | Sen (%) | Spe (%) | ACC | F1 score |
| --- | --- | --- | --- | --- | --- |
| 6 signals | 0.861  (0.816-0.907) | 0.825 | 0.896 | 0.861 | 0.854 |
| 145.9673 | 0.728  (0.669-0.787) | 0.814 | 0.726 | 0.740 | 0.753 |
| 147.975 | 0.68287  (0.621-0.745) | 0.843 | 0.669 | 0.698 | 0.733 |
| 151.0187 | 0.80237  (0.751-0.853) | 0.737 | 0.849 | 0.792 | 0.776 |
| 166.9912 | 0.83719  (0.788-0.886) | 0.779 | 0.868 | 0.820 | 0.813 |
| 208.0619 | 0.78236  (0.728-0.836) | 0.694 | 0.840 | 0.774 | 0.751 |
| 225.0456 | 0.77725  (0.722-0.832) | 0.716 | 0.879 | 0.774 | 0.758 |

Table S9. Information of external validation cohort samples.

| Number | Sex | Age | BMI | Center |
| --- | --- | --- | --- | --- |
| 1 | female | 49 | 24.98 | A |
| 2 | female | 59 | 24.80 | A |
| 3 | male | 37 | 32.10 | A |
| 4 | female | 57 | 24.02 | A |
| 5 | male | 54 | 26.42 | A |
| 6 | male | 35 | 26.89 | A |
| 7 | male | 38 | 26.22 | A |
| 8 | female | 39 | 21.72 | A |
| 9 | female | 48 | 26.67 | A |
| 10 | female | 26 | 20.03 | A |
| 11 | male | 43 | 19.03 | A |
| 12 | female | 36 | 20.58 | A |
| 13 | male | 50 | 26.57 | A |
| 14 | female | 44 | 24.03 | A |
| 15 | female | 46 | 23.33 | A |
| 16 | male | 40 | 24.22 | A |
| 17 | female | 49 | 25.77 | A |
| 18 | female | 57 | 22.04 | A |
| 19 | female | 60 | 22.86 | A |
| 20 | female | 50 | 24.97 | A |
| 21 | female | 57 | 21.68 | A |
| 22 | female | 58 | 24.52 | A |
| 23 | female | 50 | 20.81 | A |
| 24 | female | 32 | 19.10 | A |
| 25 | female | 49 | 23.53 | A |
| 26 | female | 47 | 18.43 | A |
| 27 | male | 29 | 27.10 | A |
| 28 | male | 36 | 27.68 | A |
| 29 | female | 41 | 23.63 | A |
| 30 | female | 43 | 23.88 | A |
| 31 | female | 48 | 22.22 | A |
| 32 | female | 55 | 27.30 | B |
| 33 | female | 41 | 25.20 | B |
| 34 | female | 53 | 30.80 | B |
| 35 | female | 44 | 22.31 | B |
| 36 | male | 46 | 22.80 | B |
| 37 | male | 46 | 27.20 | B |
| 38 | male | 47 | 23.00 | B |
| 39 | male | 54 | 31.20 | B |
| 40 | female | 56 | 32.00 | B |
| 41 | male | 50 | 32.70 | B |
| 42 | male | 47 | 21.30 | C |
| 43 | male | 49 | 25.28 | C |
| 44 | female | 59 | 32.41 | C |
| 45 | female | 46 | 22.58 | C |
| 46 | female | 15 | 17.63 | C |
| 47 | female | 45 | 20.89 | C |
| 48 | female | 49 | 23.01 | C |
| 49 | female | 48 | 22.27 | C |

A: Huashan Hospital

B: Liaocheng People’s Hospital

C: The First Affiliated Hospital of Fujian Medical University Hospital

Table S10. The pathway information of the potential biomarkers for MMD diagnosis.

| Pathway Name | P-value | FDR | Impact |
| --- | --- | --- | --- |
| Taurine and hypotaurine metabolism | 0.0202 | 0.8372 | 0.4286 |
| Vitamin B6 metabolism | 0.0227 | 0.8372 | 0.0784 |
| Nicotinate and nicotinamide metabolism | 0.0376 | 0.8372 | 0.0 |
| Pantothenate and CoA biosynthesis | 0.0499 | 0.8372 | 0.0 |
| beta-Alanine metabolism | 0.0523 | 0.8372 | 0.0 |
| Pyrimidine metabolism | 0.0955 | 1.0 | 0.0526 |
| Primary bile acid biosynthesis | 0.1119 | 1.0 | 0.0076 |

Table S11. Summary of 92 MMD patients with cognitive impairment.

| Number | Sex | Age | Group | Cognitive diagnosis |
| --- | --- | --- | --- | --- |
| 1 | male | 46 | 3 | VaD |
| 2 | female | 29 | 3 | VaD |
| 3 | female | 39 | 3 | VaD |
| 4 | male | 31 | 2 | VaD |
| 5 | female | 65 | 3 | VaD |
| 6 | female | 67 | 2 | mild VCI |
| 7 | male | 58 | 3 | VaD |
| 8 | female | 44 | 3 | mild VCI |
| 9 | male | 58 | 3 | mild VCI |
| 10 | female | 46 | 1 | mild VCI |
| 11 | female | 42 | 3 | mild VCI |
| 12 | male | 46 | 1 | mild VCI |
| 13 | female | 51 | 3 | mild VCI |
| 14 | female | 60 | 3 | mild VCI |
| 15 | female | 52 | 1 | mild VCI |
| 16 | female | 58 | 3 | mild VCI |
| 17 | male | 58 | 3 | mild VCI |
| 18 | male | 58 | 3 | mild VCI |
| 19 | male | 43 | 3 | mild VCI |
| 20 | female | 54 | 3 | mild VCI |
| 21 | male | 64 | 3 | mild VCI |
| 22 | male | 47 | 3 | mild VCI |
| 23 | male | 51 | 3 | mild VCI |
| 24 | male | 57 | 3 | mild VCI |
| 25 | female | 49 | 1 | mild VCI |
| 26 | female | 39 | 2 | mild VCI |
| 27 | male | 40 | 2 | mild VCI |
| 28 | female | 54 | 3 | mild VCI |
| 29 | male | 27 | 1 | mild VCI |
| 30 | female | 52 | 3 | mild VCI |
| 31 | male | 54 | 2 | mild VCI |
| 32 | female | 58 | 3 | mild VCI |
| 33 | female | 46 | 3 | mild VCI |
| 34 | male | 28 | 3 | mild VCI |
| 35 | male | 36 | 3 | mild VCI |
| 36 | female | 50 | 3 | mild VCI |
| 37 | female | 59 | 3 | mild VCI |
| 38 | female | 51 | 3 | mild VCI |
| 39 | female | 39 | 2 | mild VCI |
| 40 | female | 44 | 3 | mild VCI |
| 41 | female | 57 | 3 | mild VCI |
| 42 | male | 58 | 3 | mild VCI |
| 43 | male | 42 | 3 | mild VCI |
| 44 | male | 49 | 3 | mild VCI |
| 45 | male | 53 | 1 | VaD |
| 46 | male | 65 | 3 | VaD |
| 47 | female | 32 | 2 | normal |
| 48 | male | 39 | 3 | VaD |
| 49 | male | 49 | 3 | mild VCI |
| 50 | male | 39 | 1 | normal |
| 51 | male | 43 | 3 | mild VCI |
| 52 | male | 59 | 3 | mild VCI |
| 53 | male | 46 | 1 | normal |
| 54 | male | 47 | 3 | normal |
| 55 | female | 46 | 3 | normal |
| 56 | female | 22 | 1 | normal |
| 57 | female | 37 | 3 | mild VCI |
| 58 | male | 50 | 1 | normal |
| 59 | male | 54 | 1 | mild VCI |
| 60 | male | 57 | 3 | mild VCI |
| 61 | female | 59 | 1 | mild VCI |
| 62 | male | 52 | 3 | mild VCI |
| 63 | female | 49 | 1 | normal |
| 64 | male | 24 | 1 | normal |
| 65 | female | 45 | 2 | mild VCI |
| 66 | female | 64 | 3 | mild VCI |
| 67 | female | 29 | 3 | normal |
| 68 | female | 38 | 3 | normal |
| 69 | male | 32 | 1 | normal |
| 70 | male | 38 | 3 | mild VCI |
| 71 | male | 40 | 1 | normal |
| 72 | female | 25 | 3 | normal |
| 73 | male | 43 | 1 | normal |
| 74 | female | 50 | 1 | VaD |
| 75 | male | 51 | 3 | normal |
| 76 | female | 41 | 1 | normal |
| 77 | male | 40 | 3 | mild VCI |
| 78 | male | 35 | 1 | normal |
| 79 | female | 18 | 3 | normal |
| 80 | male | 32 | 1 | normal |
| 81 | female | 38 | 3 | normal |
| 82 | male | 39 | 1 | normal |
| 83 | male | 40 | 2 | normal |
| 84 | male | 44 | 1 | mild VCI |
| 85 | female | 59 | 3 | mild VCI |
| 86 | male | 66 | 2 | mild VCI |
| 87 | male | 41 | 3 | normal |
| 88 | female | 38 | 1 | normal |
| 89 | male | 32 | 3 | normal |
| 90 | male | 24 | 2 | normal |
| 91 | female | 33 | 2 | normal |
| 92 | female | 40 | 2 | normal |

VaD: vascular dementia

VCI: vascular cognitive impairment

Table S12. Summary of cognitive impairment in global cognitive function and memory in 92 MMD patients.

| Sample | Global cognitive function | | Memory | | | | | | | | |
| --- | --- | --- | --- | --- | --- | --- | --- | --- | --- | --- | --- |
|  | MMSE | MES | MES-5R | AVLT-N1 | AVLT-N2 | AVLT-N3 | AVLT-N4 | AVLT-N5 | AVLT-N6 | AVLT-N7 | SDMT-90s |
| 1 | 11 | 39 | 15 | 0 | 0 | 2 | 0 | 0 | 0 | 12 | 0 |
| 2 | 11 | 77 | 40 | 1 | 1 | 1 | 1 | 1 | 1 | 13 | 1 |
| 3 | 12 | 33 | 14 | 2 | 2 | 2 | 1 | 0 | 0 | 14 | 0 |
| 4 | 12 | 36 | 16 | 2 | 2 | 3 | 0 | 0 | 0 | 19 | 2 |
| 5 | 14 | 15 | 0 | 0 | 0 | 0 | 0 | 0 | 0 | 0 | 0 |
| 6 | 17 | 41 | 22 | 2 | 2 | 4 | 0 | 0 | 0 | 12 | 0 |
| 7 | 18 | 45 | 17 | 1 | 0 | 0 | 0 | 0 | 0 | 14 | 0 |
| 8 | 19 | 38 | 19 | 2 | 3 | 2 | 2 | 2 | 2 | 17 | 3 |
| 9 | 20 | 39 | 21 | 2 | 4 | 4 | 1 | 0 | 0 | 15 | 0 |
| 10 | 20 | 81 | 38 | 4 | 4 | 7 | 4 | 3 | 3 | 21 | 3 |
| 11 | 21 | 58 | 12 | 5 | 7 | 9 | 6 | 6 | 5 | 24 | 0 |
| 12 | 21 | 68 | 27 | 2 | 3 | 5 | 2 | 1 | 1 | 18 | 3 |
| 13 | 22 | 53 | 22 | 3 | 3 | 3 | 1 | 0 | 0 | 9 | 3 |
| 14 | 22 | 66 | 35 | 1 | 5 | 8 | 9 | 10 | 9 | 24 | 5 |
| 15 | 22 | 74 | 29 | 3 | 3 | 5 | 4 | 4 | 3 | 2 | 0 |
| 16 | 23 | 61 | 31 | 1 | 2 | 4 | 3 | 3 | 2 | 18 | 1 |
| 17 | 23 | 69 | 40 | 3 | 4 | 4 | 1 | 0 | 1 | 19 | 0 |
| 18 | 23 | 72 | 33 | 4 | 2 | 4 | 1 | 0 | 0 | 17 | 2 |
| 19 | 23 | 74 | 32 | 3 | 5 | 6 | 4 | 4 | 5 | 22 | 2 |
| 20 | 23 | 76 | 35 | 4 | 8 | 6 | 6 | 7 | 7 | 23 | 3 |
| 21 | 24 | 45 | 25 | 0 | 2 | 4 | 2 | 2 | 1 | 20 | 1 |
| 22 | 24 | 74 | 36 | 0 | 2 | 1 | 0 | 0 | 0 | 21 | 1 |
| 23 | 24 | 75 | 32 | 4 | 4 | 5 | 3 | 2 | 2 | 2 | 0 |
| 24 | 24 | 90 | 42 | 2 | 4 | 3 | 5 | 1 | 2 | 11 | 7 |
| 25 | 25 | 43 | 15 | 0 | 0 | 0 | 0 | 0 | 0 | 20 | 3 |
| 26 | 25 | 59 | 27 | 2 | 3 | 4 | 4 | 3 | 3 | 13 | 6 |
| 27 | 25 | 80 | 37 | 4 | 4 | 5 | 3 | 3 | 3 | 20 | 2 |
| 28 | 25 | 83 | 40 | 4 | 8 | 9 | 7 | 5 | 4 | 23 | 5 |
| 29 | 26 | 66 | 28 | 2 | 6 | 7 | 5 | 5 | 2 | 23 | 6 |
| 30 | 26 | 73 | 36 | 1 | 3 | 4 | 0 | 0 | 0 | 18 | 0 |
| 31 | 26 | 78 | 36 | 2 | 5 | 5 | 5 | 5 | 5 | 22 | 0 |
| 32 | 26 | 80 | 36 | 5 | 4 | 6 | 4 | 3 | 4 | 18 | 9 |
| 33 | 27 | 52 | 30 | 3 | 4 | 7 | 5 | 3 | 2 | 18 | 5 |
| 34 | 27 | 79 | 43 | 3 | 6 | 9 | 8 | 8 | 8 | 22 | 1 |
| 35 | 27 | 83 | 39 | 3 | 4 | 7 | 5 | 3 | 3 | 20 | 3 |
| 36 | 28 | 70 | 42 | 2 | 4 | 6 | 4 | 3 | 3 | 22 | 4 |
| 37 | 28 | 76 | 36 | 4 | 5 | 4 | 1 | 1 | 1 | 17 | 2 |
| 38 | 28 | 77 | 34 | 4 | 5 | 7 | 3 | 3 | 3 | 20 | 2 |
| 39 | 28 | 79 | 34 | 2 | 5 | 7 | 6 | 6 | 3 | 19 | 1 |
| 40 | 28 | 87 | 41 | 8 | 10 | 9 | 8 | 8 | 8 | 23 | 1 |
| 41 | 28 | 94 | 47 | 4 | 5 | 7 | 3 | 2 | 3 | 20 | 0 |
| 42 | 29 | 86 | 38 | 2 | 3 | 6 | 3 | 3 | 2 | 19 | 2 |
| 43 | 30 | 62 | 36 | 3 | 3 | 4 | 0 | 0 | 0 | 13 | 1 |
| 44 | 30 | 84 | 38 | 4 | 4 | 8 | 6 | 7 | 2 | 22 | 5 |
| 45 | 3 | 0 | 0 | 0 | 0 | 0 | 0 | 0 | 0 | 0 | 0 |
| 46 | 11 | 45 | 17 | 2 | 3 | 2 | 0 | 2 | 2 | 15 | 0 |
| 47 | 26 | 91 | 46 | 6 | 4 | 7 | 3 | 4 | 4 | 19 | 0 |
| 48 | 17 | 47 | 18 | 2 | 3 | 3 | 0 | 0 | 0 | 18 | 0 |
| 49 | 19 | 60 | 26 | 2 | 5 | 4 | 0 | 0 | 0 | 14 | 0 |
| 50 | 27 | 80 | 33 | 8 | 11 | 10 | 11 | 10 | 11 | 23 | 4 |
| 51 | 20 | 54 | 26 | 4 | 3 | 1 | 0 | 0 | 1 | 18 | 0 |
| 52 | 20 | 56 | 34 | 2 | 2 | 3 | 2 | 2 | 0 | 19 | 0 |
| 53 | 27 | 94 | 48 | 3 | 5 | 7 | 7 | 7 | 10 | 23 | 5 |
| 54 | 27 | 95 | 49 | 7 | 10 | 11 | 10 | 10 | 9 | 24 | 4 |
| 55 | 27 | 98 | 48 | 6 | 6 | 9 | 7 | 5 | 7 | 24 | 1 |
| 56 | 28 | 69 | 27 | 4 | 3 | 4 | 5 | 5 | 5 | 18 | 1 |
| 57 | 22 | 68 | 29 | 3 | 4 | 3 | 0 | 0 | 0 | 17 | 0 |
| 58 | 28 | 79 | 42 | 5 | 5 | 6 | 6 | 6 | 5 | 23 | 5 |
| 59 | 25 | 70 | 35 | 4 | 4 | 4 | 2 | 1 | 0 | 12 | 2 |
| 60 | 25 | 75 | 38 | 5 | 6 | 7 | 4 | 5 | 5 | 20 | 2 |
| 61 | 25 | 79 | 45 | 4 | 2 | 3 | 4 | 3 | 1 | 18 | 4 |
| 62 | 27 | 89 | 42 | 0 | 5 | 5 | 3 | 4 | 2 | 19 | 1 |
| 63 | 28 | 89 | 43 | 2 | 5 | 9 | 8 | 10 | 8 | 22 | 9 |
| 64 | 28 | 97 | 47 | 5 | 8 | 9 | 7 | 6 | 5 | 23 | 5 |
| 65 | 28 | 85 | 41 | 4 | 6 | 6 | 7 | 6 | 4 | 22 | 6 |
| 66 | 29 | 64 | 32 | 4 | 6 | 7 | 3 | 3 | 0 | 23 | 2 |
| 67 | 29 | 84 | 34 | 3 | 8 | 9 | 9 | 10 | 9 | 24 | 9 |
| 68 | 29 | 84 | 37 | 5 | 6 | 6 | 5 | 6 | 5 | 18 | 4 |
| 69 | 29 | 92 | 46 | 4 | 6 | 7 | 5 | 4 | 3 | 20 | 3 |
| 70 | 29 | 97 | 49 | 10 | 10 | 9 | 7 | 6 | 4 | 22 | 0 |
| 71 | 29 | 93 | 46 | 9 | 12 | 12 | 12 | 11 | 12 | 24 | 4 |
| 72 | 29 | 96 | 46 | 5 | 8 | 10 | 9 | 9 | 9 | 24 | 6 |
| 73 | 29 | 97 | 49 | 7 | 9 | 6 | 7 | 8 | 7 | 22 | 9 |
| 74 | 18 | 53 | 18 | 1 | 2 | 3 | 0 | 0 | 0 | 22 | 0 |
| 75 | 30 | 93 | 48 | 5 | 7 | 11 | 9 | 9 | 11 | 24 | 7 |
| 76 | 30 | 96 | 47 | 6 | 7 | 7 | 5 | 5 | 5 | 20 | 5 |
| 77 | 25 | 81 | 35 | 4 | 7 | 7 | 4 | 4 | 4 | 23 | 4 |
| 78 | 30 | 96 | 49 | 7 | 6 | 6 | 7 | 6 | 6 | 23 | 7 |
| 79 | 30 | 97 | 48 | 7 | 7 | 10 | 8 | 8 | 8 | 24 | 0 |
| 80 | 28 | 88 | 46 | 3 | 6 | 7 | 3 | 4 | 5 | 23 | 7 |
| 81 | 29 | 96 | 47 | 5 | 10 | 12 | 11 | 11 | 12 | 24 | 6 |
| 82 | 29 | 96 | 49 | 3 | 7 | 6 | 4 | 4 | 2 | 19 | 5 |
| 83 | 30 | 99 | 49 | 5 | 6 | 7 | 7 | 6 | 7 | 24 | 3 |
| 84 | 25 | 88 | 40 | 3 | 5 | 4 | 2 | 1 | 2 | 2 | 49 |
| 85 | 26 | 75 | 30 | 2 | 3 | 3 | 3 | 3 | 3 | 21 | 0 |
| 86 | 28 | 65 | 26 | 3 | 2 | 3 | 2 | 2 | 0 | 12 | 0 |
| 87 | 27 | 94 | 47 | 5 | 5 | 6 | 5 | 4 | 3 | 20 | 6 |
| 88 | 28 | 79 | 34 | 2 | 5 | 7 | 6 | 6 | 3 | 19 | 1 |
| 89 | 28 | 89 | 43 | 10 | 12 | 12 | 9 | 12 | 12 | 24 | 2 |
| 90 | 28 | 95 | 48 | 10 | 11 | 12 | 10 | 11 | 12 | 24 | 5 |
| 91 | 28 | 96 | 46 | 3 | 6 | 11 | 8 | 8 | 10 | 23 | 9 |
| 92 | 30 | 96 | 47 | 6 | 8 | 12 | 12 | 12 | 12 | 24 | 4 |

MMSE: Mini-Mental State Examination

MES: Memory and Executive Screening

MES-EX: MES Executive Part

MES-5R: MES Memory Part

AVLT: Chinese Auditory Verbal Learning Test

AVLT N1-N3: AVLT short-term recall 1 to 3

AVLT N4: AVLT short delay recall

AVLT N5: AVLT long delay recall

AVLT N6: AVLT cue recall

AVLT N7: AVLT recognition

SDMT: Symbol Digit Modalities Test

SDMT-90s: SDMT Accidental Recall after 90 seconds

TMT-A: Trial Making Test A

TMT-B: Trial Making Test B

TMT-B 1min: TMT-B Reaching Numbers in 1 minute

VFT: Verbal Fluency Test

CDT: Clock Drawing Test

CFT: Rey-Osterrieth Complex Figure Test

BNT: Boston Naming Test

Table S13. Summary of cognitive impairment in attention, executive function, visuospital function and language in 92 MMD patients.

| Sample | Attention | | Executive function | | | | Visuospital function | | Language | | |
| --- | --- | --- | --- | --- | --- | --- | --- | --- | --- | --- | --- |
|  | SDMT | TMT-A(s) | MES-EX | TMT-B(s) | TMT-B1min | VFT-alternation | CDT | CFT | BNT | VFT-animal | VFT-vegetable |
| 1 | 4 | 150 | 24 | 334 | 4 | 2 | 0 | 9 | 14 | 0 | 3 |
| 2 | 26 | 88 | 37 | 185 | 8 | 6 | 17 | 25 | 7 | 2 | 3 |
| 3 | 0 | 240 | 19 | 360 | 0 | 4 | 0 | 0 | 14 | 4 | 5 |
| 4 | 19 | 145 | 20 | 391 | 6 | 6 | 9 | 6 | 24 | 6 | 5 |
| 5 | 0 | 240 | 15 | 360 | 0 | 0 | 0 | 0 | 10 | 0 | 3 |
| 6 | 1 | 124 | 19 | 339 | 5 | 11 | 1 | 7 | 20 | 10 | 10 |
| 7 | 0 | 240 | 28 | 360 | 0 | 13 | 0 | 6 | 11 | 17 | 14 |
| 8 | 32 | 57 | 19 | 140 | 9 | 10 | 15 | 28 | 17 | 15 | 11 |
| 9 | 11 | 126 | 18 | 236 | 8 | 6 | 10 | 11 | 18 | 7 | 5 |
| 10 | 23 | 70 | 43 | 131 | 16 | 10 | 0 | 27 | 18 | 5 | 8 |
| 11 | 0 | 88 | 46 | 191 | 7 | 8 | 0 | 0 | 22 | 12 | 14 |
| 12 | 20 | 72 | 41 | 196 | 9 | 6 | 16 | 34 | 22 | 8 | 12 |
| 13 | 19 | 77 | 31 | 142 | 13 | 6 | 16 | 17 | 22 | 5 | 3 |
| 14 | 21 | 55 | 31 | 160 | 12 | 13 | 6 | 34 | 19 | 11 | 12 |
| 15 | 22 | 67 | 45 | 112 | 16 | 10 | 17 | 18 | 22 | 9 | 11 |
| 16 | 11 | 71 | 30 | 207 | 7 | 15 | 12 | 25 | 22 | 15 | 13 |
| 17 | 19 | 67 | 29 | 210 | 7 | 3 | 19 | 32 | 19 | 9 | 3 |
| 18 | 26 | 49 | 39 | 126 | 11 | 14 | 25 | 35 | 21 | 11 | 12 |
| 19 | 33 | 51 | 42 | 121 | 11 | 5 | 24 | 34 | 26 | 11 | 7 |
| 20 | 20 | 97 | 41 | 172 | 9 | 9 | 14 | 26 | 19 | 10 | 7 |
| 21 | 10 | 100 | 20 | 205 | 7 | 11 | 21 | 10 | 19 | 8 | 8 |
| 22 | 21 | 95 | 38 | 145 | 13 | 7 | 26 | 24 | 19 | 9 | 5 |
| 23 | 21 | 65 | 43 | 155 | 9 | 10 | 26 | 32 | 20 | 11 | 9 |
| 24 | 52 | 48 | 48 | 76 | 20 | 8 | 25 | 27 | 25 | 17 | 16 |
| 25 | 22 | 67 | 28 | 208 | 8 | 11 | 14 | 33 | 12 | 9 | 7 |
| 26 | 45 | 50 | 32 | 116 | 14 | 11 | 22 | 33 | 20 | 14 | 12 |
| 27 | 50 | 34 | 43 | 102 | 15 | 12 | 17 | 35 | 22 | 13 | 12 |
| 28 | 20 | 89 | 43 | 176 | 7 | 13 | 22 | 21 | 20 | 12 | 14 |
| 29 | 38 | 59 | 38 | 131 | 12 | 14 | 26 | 24 | 23 | 13 | 12 |
| 30 | 31 | 70 | 37 | 135 | 11 | 12 | 15 | 30 | 17 | 17 | 10 |
| 31 | 15 | 211 | 42 | 245 | 6 | 10 | 14 | 11 | 18 | 13 | 10 |
| 32 | 41 | 45 | 44 | 112 | 14 | 10 | 29 | 35 | 21 | 8 | 7 |
| 33 | 32 | 70 | 22 | 178 | 12 | 6 | 28 | 26 | 22 | 16 | 9 |
| 34 | 29 | 73 | 36 | 143 | 13 | 10 | 20 | 6 | 19 | 12 | 7 |
| 35 | 38 | 52 | 44 | 110 | 14 | 8 | 26 | 36 | 29 | 21 | 9 |
| 36 | 32 | 107 | 28 | 151 | 11 | 8 | 15 | 14 | 19 | 12 | 7 |
| 37 | 21 | 62 | 40 | 132 | 13 | 9 | 17 | 25 | 19 | 10 | 6 |
| 38 | 33 | 53 | 43 | 119 | 15 | 10 | 17 | 26 | 16 | 10 | 9 |
| 39 | 35 | 50 | 45 | 120 | 13 | 10 | 16 | 27 | 14 | 10 | 9 |
| 40 | 45 | 38 | 46 | 149 | 10 | 14 | 21 | 28 | 28 | 17 | 11 |
| 41 | 35 | 58 | 47 | 154 | 11 | 6 | 15 | 32 | 19 | 9 | 11 |
| 42 | 48 | 33 | 48 | 87 | 19 | 12 | 28 | 30 | 25 | 16 | 11 |
| 43 | 30 | 66 | 26 | 151 | 9 | 2 | 16 | 22 | 22 | 8 | 7 |
| 44 | 45 | 42 | 46 | 126 | 11 | 12 | 28 | 28 | 20 | 14 | 9 |
| 45 | 0 | 240 | 0 | 360 | 0 | 0 | 0 | 0 | 0 | 0 | 0 |
| 46 | 0 | 64 | 28 | 360 | 0 | 6 | 0 | 0 | 15 | 10 | 11 |
| 47 | 45 | 40 | 45 | 86 | 17 | 17 | 12 | 30 | 20 | 12 | 9 |
| 48 | 18 | 240 | 29 | 360 | 0 | 6 | 0 | 2 | 16 | 7 | 6 |
| 49 | 20 | 61 | 34 | 162 | 11 | 15 | 0 | 0 | 17 | 12 | 10 |
| 50 | 44 | 51 | 47 | 116 | 13 | 12 | 26 | 32 | 24 | 14 | 11 |
| 51 | 21 | 66 | 28 | 130 | 12 | 6 | 0 | 8 | 15 | 8 | 6 |
| 52 | 17 | 70 | 22 | 219 | 6 | 8 | 9 | 27 | 21 | 10 | 11 |
| 53 | 43 | 38 | 46 | 103 | 17 | 10 | 21 | 27 | 25 | 11 | 11 |
| 54 | 38 | 68 | 46 | 120 | 13 | 10 | 8 | 34 | 28 | 16 | 13 |
| 55 | 52 | 38 | 50 | 79 | 20 | 12 | 22 | 36 | 25 | 10 | 10 |
| 56 | 49 | 50 | 42 | 114 | 14 | 16 | 24 | 34 | 25 | 23 | 14 |
| 57 | 35 | 84 | 39 | 246 | 8 | 9 | 15 | 29 | 24 | 10 | 10 |
| 58 | 25 | 59 | 37 | 152 | 11 | 12 | 21 | 28 | 20 | 10 | 4 |
| 59 | 14 | 140 | 35 | 263 | 7 | 4 | 17 | 27 | 19 | 10 | 7 |
| 60 | 32 | 67 | 37 | 177 | 7 | 12 | 21 | 28 | 21 | 11 | 10 |
| 61 | 21 | 68 | 34 | 158 | 11 | 13 | 5 | 2 | 15 | 17 | 12 |
| 62 | 24 | 56 | 47 | 150 | 9 | 8 | 5 | 36 | 23 | 7 | 9 |
| 63 | 55 | 37 | 46 | 133 | 13 | 13 | 9 | 34 | 24 | 14 | 12 |
| 64 | 49 | 35 | 50 | 95 | 14 | 10 | 18 | 27 | 26 | 14 | 13 |
| 65 | 28 | 50 | 44 | 108 | 13 | 16 | 21 | 32 | 19 | 12 | 13 |
| 66 | 23 | 79 | 32 | 166 | 9 | 10 | 29 | 22 | 20 | 13 | 15 |
| 67 | 65 | 40 | 50 | 88 | 20 | 16 | 27 | 34 | 26 | 23 | 20 |
| 68 | 42 | 36 | 47 | 97 | 14 | 14 | 22 | 36 | 23 | 17 | 16 |
| 69 | 35 | 53 | 46 | 191 | 10 | 9 | 28 | 29 | 25 | 17 | 12 |
| 70 | 60 | 23 | 48 | 84 | 14 | 9 | 30 | 33 | 22 | 12 | 9 |
| 71 | 54 | 39 | 47 | 70 | 23 | 20 | 17 | 35 | 27 | 27 | 19 |
| 72 | 48 | 29 | 50 | 72 | 22 | 12 | 19 | 35 | 28 | 17 | 10 |
| 73 | 62 | 49 | 48 | 80 | 20 | 16 | 28 | 35 | 25 | 21 | 13 |
| 74 | 0 | 103 | 35 | 166 | 10 | 9 | 0 | 18 | 13 | 12 | 19 |
| 75 | 50 | 51 | 45 | 87 | 18 | 21 | 30 | 35 | 27 | 19 | 18 |
| 76 | 56 | 45 | 49 | 82 | 17 | 19 | 28 | 35 | 26 | 16 | 18 |
| 77 | 39 | 50 | 46 | 100 | 13 | 7 | 22 | 31 | 23 | 19 | 13 |
| 78 | 65 | 26 | 47 | 64 | 24 | 17 | 30 | 36 | 29 | 17 | 16 |
| 79 | 46 | 46 | 49 | 124 | 13 | 13 | 26 | 31 | 27 | 13 | 9 |
| 80 | 40 | 37 | 42 | 75 | 22 | 12 | 15 | 33 | 23 | 14 | 11 |
| 81 | 54 | 39 | 49 | 92 | 13 | 18 | 28 | 32 | 25 | 20 | 16 |
| 82 | 35 | 48 | 47 | 134 | 14 | 14 | 27 | 30 | 23 | 19 | 13 |
| 83 | 50 | 44 | 50 | 77 | 18 | 21 | 22 | 36 | 27 | 20 | 17 |
| 84 | 21 | 41 | 44 | 118 | 13 | 10 | 2 | 34 | 17 | 18 | 12 |
| 85 | 39 | 54 | 45 | 145 | 11 | 6 | 17 | 29 | 20 | 7 | 6 |
| 86 | 34 | 68 | 39 | 114 | 12 | 10 | 19 | 32 | 20 | 11 | 10 |
| 87 | 46 | 29 | 47 | 94 | 16 | 17 | 20 | 36 | 25 | 17 | 13 |
| 88 | 35 | 50 | 45 | 120 | 14 | 10 | 16 | 27 | 14 | 10 | 9 |
| 89 | 50 | 42 | 46 | 100 | 16 | 14 | 27 | 34 | 27 | 20 | 17 |
| 90 | 65 | 41 | 47 | 94 | 13 | 24 | 26 | 36 | 30 | 24 | 21 |
| 91 | 58 | 34 | 50 | 78 | 20 | 18 | 28 | 35 | 27 | 17 | 15 |
| 92 | 59 | 39 | 49 | 77 | 17 | 22 | 23 | 35 | 28 | 23 | 19 |

MMSE: Mini-Mental State Examination

MES: Memory and Executive Screening

MES-EX: MES Executive Part

MES-5R: MES Memory Part

AVLT: Chinese Auditory Verbal Learning Test

AVLT N1-N3: AVLT short-term recall 1 to 3

AVLT N4: AVLT short delay recall

AVLT N5: AVLT long delay recall

AVLT N6: AVLT cue recall

AVLT N7: AVLT recognition

SDMT: Symbol Digit Modalities Test

SDMT-90s: SDMT Accidental Recall after 90 seconds

TMT-A: Trial Making Test A

TMT-B: Trial Making Test B

TMT-B 1min: TMT-B Reaching Numbers in 1 minute

VFT: Verbal Fluency Test

CDT: Clock Drawing Test

CFT: Rey-Osterrieth Complex Figure Test

BNT: Boston Naming Test
